# Multiyear dual-isotope fingerprinting at South Asian receptor sites constrains carbon monoxide sources and enhanced oxidation

**DOI:** 10.1073/pnas.2606200123

**Published:** 2026-10-12

**Authors:** Peng Yao, Henry Holmstrand, Carina van der Veen, Maria Elena Popa, Chloe A. Brashear, Krishnakant Budhavant, Mohanan Remani Manoj, Joakim Romson, Abdus Salam, Thomas Röckmann, Örjan Gustafsson

**Affiliations:** ^a^ Department of Environmental Science and Bolin Centre for Climate Research, Stockholm University, Stockholm 10691, Sweden; ^b^ Institute for Marine and Atmospheric Research Utrecht, Utrecht University, Utrecht 3584CC, The Netherlands; ^c^Maldives Climate Observatory-Hanimaadhoo, Maldives Meteorological Services, H. Dh. Hanimaadhoo 02020, Maldives; ^d^Divecha Centre for Climate Change, Indian Institute of Science, Bangalore 560012, India; ^e^ Department of Chemistry, University of Dhaka, Dhaka 1000, Bangladesh

**Keywords:** CO, isotope, combustion, atmospheric oxidation, source apportionment

## Abstract

Carbon monoxide (CO) is an indirect short-lived climate forcer with uncertainties both in sources and its role in atmospheric oxidation. Based on nine winter-long dual-isotope campaigns at two South Asian receptor sites intercepting the continental outflow, we quantified CO source contributions and emission–sink dynamics. Combustion accounts for 68 to 74% of South Asia regional CO (including 34 to 37% from biomass burning) with secondary atmospheric oxidation contributing 26 to 32% (dominated by oxidation of nonmethane volatile organic compounds (NMVOCs) at 21 to 26%, with methane oxidation contributing 5.5 to 6.4%). These isotope-observational constraints suggest a twice higher role for atmospheric oxidation than in model estimates. Spatially, the absolute contributions of both primary and secondary CO decrease from the Indo-Gangetic Plain to the northern Indian Ocean, while the relative contribution of oxidation-derived CO increases, indicating enhanced secondary CO production near source regions. Observation-model comparison suggests that transport from the continental areas dominates CO over adjacent oceanic regions, with minor local production. During the COVID-19 emission perturbation, combustion-derived CO fell sharply in absolute terms, NMVOC-derived CO rose, and CH_4_-derived CO remained stable, providing an observational constraint on the sensitivity of atmospheric oxidation to emission changes. Our results reveal a significant contribution of CO from atmospheric oxidation in South Asia, calling for a reassessment of regional emission–oxidation coupling in models and of mitigation strategies.

## Significance

Carbon monoxide (CO) regulates atmospheric oxidation and the lifetime of multiple climate forcers. Current understanding of its sources largely draws on emission inventories, satellite observations, and inversion models, which provide valuable but indirectly constrained estimates. Using multiyear dual-isotope observations at two South Asian background sites, we deliver an independent, observation-based, top–down constraint on CO sources and oxidation. We find that oxidation-derived CO is more than twice as large as suggested by model-based estimates, indicating a stronger regional oxidative capacity. Continental emissions dominate CO even over adjacent oceans, highlighting the role of long-range transport. Changes during the COVID-19 lockdown illustrate how emission reductions reshape atmospheric chemistry. These results provide observational constraints to refine models and inform mitigation strategies.

Carbon monoxide (CO) is a key reactive trace gas with an atmospheric lifetime of 1 to 4 months (1), playing a central role in atmospheric chemistry and the short-lived climate forcers (SLCFs) system (2). Through reaction with hydroxyl radical (OH), CO influences the oxidation capacity of the atmosphere, competes with methane (CH_4_) and nonmethane volatile organic compounds (NMVOCs), and indirectly affects ozone (O_3_) formation and the lifetimes of other SLCFs (3). Globally, CO is responsible for ~40% of OH loss, making it a key regulator of atmospheric oxidation (3). The IPCC attributed 0.44 W m^−2^ of effective radiative forcing (2) to CO and NMVOCs combined, comparable to CH_4_ (0.54 W m^−2^) and O_3_ (0.47 W m^−2^). At high concentrations, CO is also toxic and co-occurs with harmful air pollutants such as aerosols and O_3_ (4, 5), contributing to poor air quality and health risks.

CO is emitted from primary combustion sources (e.g., fossil fuel and biomass burning) and is also produced secondarily through oxidation of CH_4_ and NMVOCs (6). While CO sources have been mainly studied using emission inventories and inverse-atmospheric models, the model estimates of primary and secondary CO vary widely, with combustion estimates ranging from 40 to 60% globally (7–17). Models generally assume comparable CH_4_ oxidation rates when estimating CH_4_-derived CO, but contributions from NMVOC oxidation vary widely due to the poorly constrained precursor species and their oxidation pathways (18, 19). This leads to substantial variation in the partitioning of secondary CO in models, where the CH_4_ fraction varies from 53 to 80% with NMVOCs comprising the remaining share (20 to 47%) (7–17). Isotopic analysis offers an independent top–down constraint for source attribution, as demonstrated in aerosols (20, 21) and trace gas studies, including CO (22–26). Yet, challenges remain in quantifying CO sources due to limited observations of source-diagnostic isotopic composition, especially in key emission regions such as South Asia. Applying isotope-based constraints can help resolve key uncertainties, such as to quantify primary vs. secondary CO, fossil vs. biomass combustion sources, and CH_4_ vs. NMVOC, and improve support for targeted mitigation strategies.

South Asia, home to a quarter of the global population, faces rising climate and air pollution risks (20), including smog episodes, haze, heatwaves, and monsoon disruptions, with cascading effects on health, food, and water security. As one of the largest and fastest-growing emitters of greenhouse gases (13, 27), the region plays a critical role in the global climate system. However, CO source contributions in South Asia remain poorly quantified. Dedicated observational studies are scarce.

This study seeks to enhance understanding of CO emissions and atmospheric processes over South Asia through observations at the Bangladesh Climate Observatory at Bhola (BCOB), a receptor site located at the outflow of the Indo-Gangetic Plain (IGP), and the Maldives Climate Observatory at Hanimaadhoo (MCOH), a receptor site in the northern Indian Ocean, functioning as a broad footprint of the entire South Asian region. We employed dual-isotope fingerprints to classify CO sources in South Asia and applied a Markov Chain Monte Carlo (MCMC) model for quantitative source apportionment. The study focuses on variations in CO sources and atmospheric oxidation processes over recent years, with special attention to impacts during the 2019 pandemic and to transitions before and after it. The study provides detailed isotopic constraints on CO sources in South Asia, and, by analyzing both source-region and remote sites, it further resolves atmospheric processes related to transport, mixing, and sinks. These independent isotope-observational constraints are compared with existing emission inventory and model predictions for the South Asian region.

## 1. Results

### 1.1. Satellite and Ground-Based Observations of CO Emissions in South Asia.

**Fig. 1.**
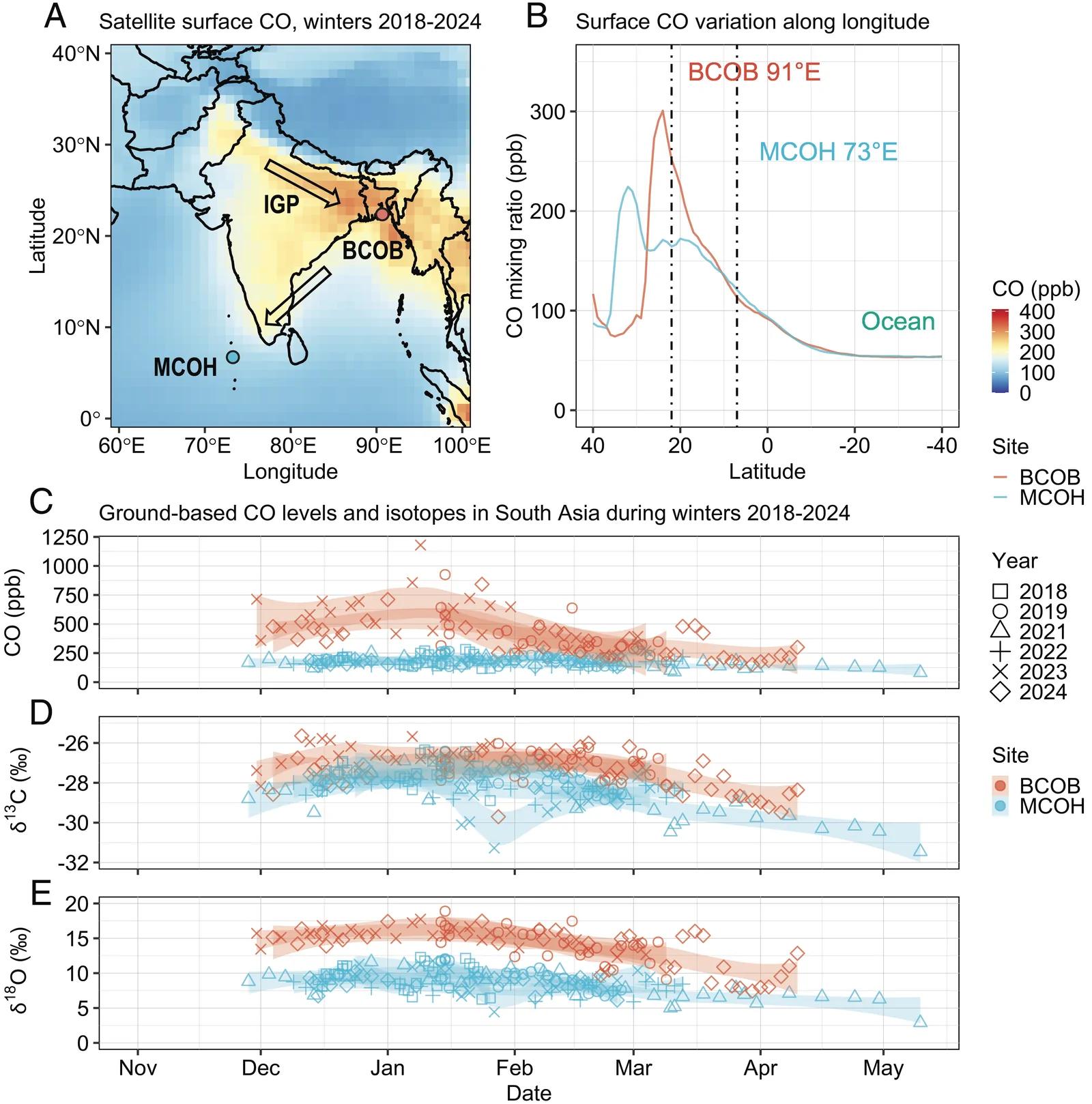
Spatial and temporal trends in CO mixing ratios and isotopic compositions across South Asia in winter seasons 2018–2024. (*A*) Satellite-derived surface CO mixing ratios (MOPITT, MOP03JM v009) over South Asia. Black arrows denote the major air mass transport directions during the winter study periods. (*B*) Latitudinal variability of surface CO mixing ratios, with dot-dash lines along the longitudes of the Bangladesh Climate Observatory at Bhola (BCOB) and the Maldives Climate Observatory at Hanimaadhoo (MCOH). Multiyear ground-based observations at BCOB and MCOH of (*C*) CO mixing ratios, (*D*) δ^13^C-CO, and (*E*) δ^18^O-CO.

Spatial and temporal trends in CO levels and isotopic compositions across South Asia in winter seasons were obtained via satellite and ground-based observations (Fig. 1). Satellite-derived surface CO mixing ratios across South Asia showed distinct spatial patterns (Fig. 1*A*), with the highest concentrations over the Bay of Bengal, followed by the IGP, the southern Deccan Plateau, and Southeast Asia. In contrast, significantly lower CO mixing ratios were seen over the Indian Ocean to the south and over the Himalaya-Tibetan Plateau to the north. The Bay of Bengal consistently exhibited the highest CO levels, likely reflecting substantial atmospheric outflow from the IGP, amended by subsequent atmospheric production. Notably, the IGP outflow site (BCOB) is situated within this high-CO region. Satellite-derived surface CO mixing ratios along the longitude corresponding to the sites BCOB (~252 ppb) and MCOH (~121 ppb) highlight the spatial variations (Fig. 1*B*). The longitudinal profiles indicated that CO mixing ratios were significantly elevated over the South Asian continent, decreasing rapidly with distance over the ocean before reaching regional oceanic background levels (down to 50 to 60 ppb).

Ground-based in situ observations (Fig. 1*C*) revealed higher CO mixing ratios at both BCOB (median = 366 ppb; 95% CI: 323 to 418 ppb) and MCOH (median = 178 ppb, 95% CI: 165 to 183 ppb) than satellite estimates, likely due to broader vertical averaging and/or lower spatial resolution of satellite retrievals. Ground-based CO mixing ratios at BCOB were consistently higher than those at MCOH, occasionally surpassing 1,000 ppb, due to its proximity to high-pollution sources in South Asia and the location at the outflow of the pollution-integrating IGP. In contrast, MCOH exhibited lower and more stable CO mixing ratios, mostly in the range 100 to 200 ppb. However, these are also significantly above Indian Ocean background levels, underscoring the strategic location and role of MCOH as an outflow receptor site for broader South Asian emissions. Recent studies on CO mixing ratios in South Asia reported similarly elevated levels (28, 29), consistent with ground-based observations in the current long-term study.

Multiyear isotopic observations (Fig. 1 *D* and *E*) showed distinct site-specific signatures. In general, CO isotopes at BCOB displayed greater variability due to its proximity to emission sources, while CO isotopic compositions at MCOH were more stable, as were the CO mole fractions. The δ^13^C values of CO varied modestly, with BCOB showing slightly more enriched values than MCOH, reflecting its closer vicinity to combustion sources. The δ^18^O values exhibited more pronounced differences at BCOB, with higher δ^18^O enrichment likely due to combustion-related inputs. These isotopic values and differences captured both CO source characteristics and atmospheric processes.

### 1.2. Isotopic Insights into CO Sources and Atmospheric Processes.

**Fig. 2.**
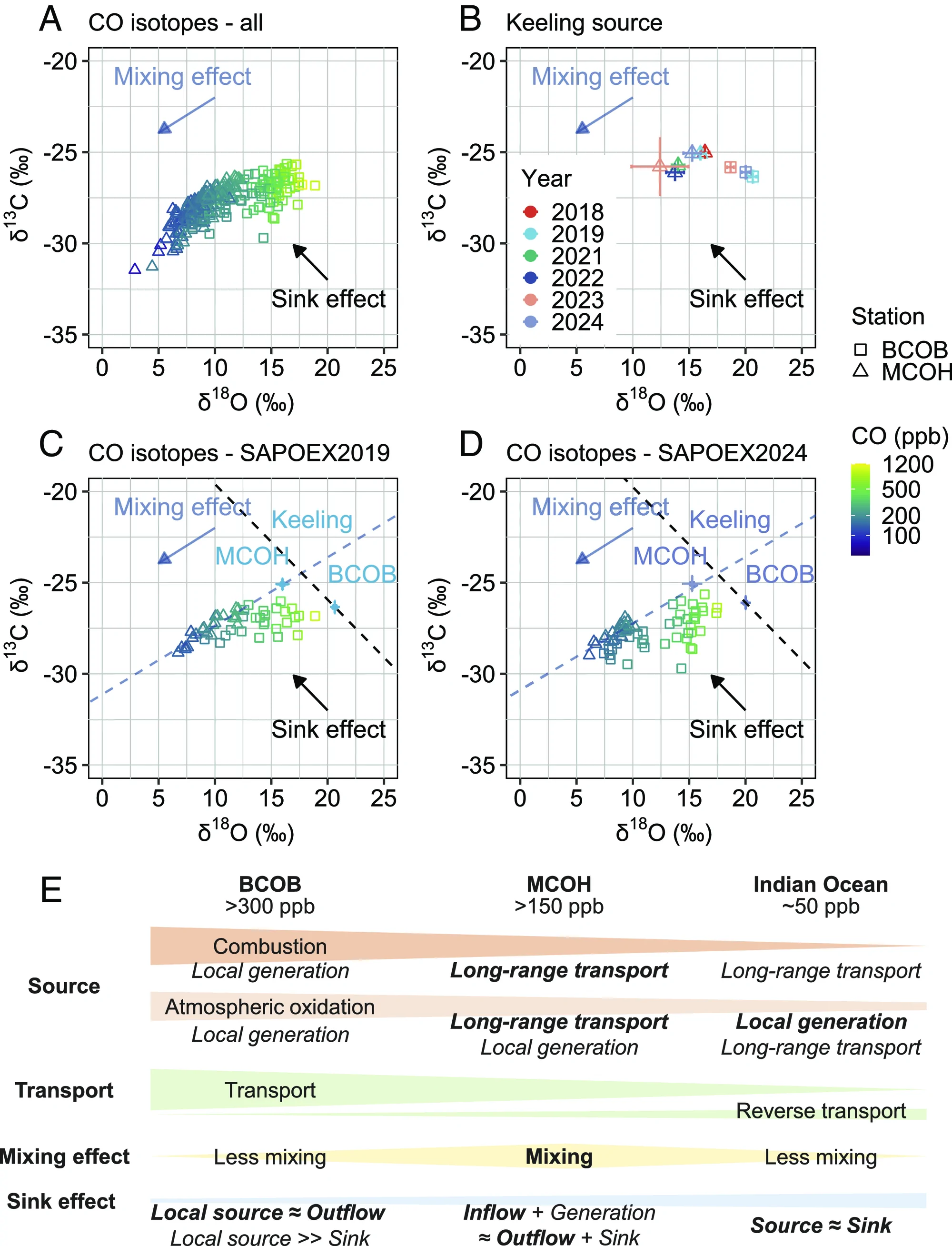
Isotopic compositions and source analysis of CO at outflow (BCOB) and receptor (MCOH) sites in South Asia. (*A*) Dual CO isotopic compositions (δ^13^C and δ^18^O) at BCOB and MCOH. (*B*) Isotopic compositions of the regional representative source inferred from Keeling plot intercepts. Campaign-specific isotopic variations during SAPOEX (South Asian Pollution Experiment) field campaigns: (*C*) 2019 and (*D*) 2024, with markers indicating Keeling plot-derived source signatures. The black arrows indicate the kinetic isotopic fractionation caused by the CO sink, determined by reaction rate ratios (*SI Appendix*, Table S8). The black dashed lines refer to the direction of the sink effect from BCOB to MCOH transport. The blue dashed lines refer to fitted lines of δ^18^O versus δ^13^C in MCOH, indicating the mixing effect. (*E*) Key processes governing CO isotopic compositions: source emissions, transport, mixing, and sinks. The dominant sources are combustion emissions, confined to land but transported over the ocean, and atmospheric oxidation, which occurs over both land and ocean. Transport involves both the export of source-derived CO toward the background region and occasional reverse transport. The mixing effect mainly reflects the combination of source CO from the IGP region, secondary CO, and local background CO, both at the observation region and along the transport pathway. The sink effect becomes increasingly pronounced along the transport pathway from the source region to the Indian Ocean background, eventually reaching a steady state.

To disentangle the source signals and atmospheric processes embedded in the isotopic composition, we investigated long-range transport between two receptor sites of the South Asian outflow separated by ~2,600 km. Multiyear records of CO isotopic composition at the IGP outflow site BCOB and the Indian Ocean receptor site MCOH revealed consistent spatial patterns (Fig. 2). Both δ^13^C and δ^18^O values were systematically higher at BCOB than at MCOH (Fig. 2*A*). The parallel depletion of δ^13^C and δ^18^O with declining CO concentrations, both within and between BCOB and MCOH, was inconsistent with the expected kinetic isotope effect (KIE, *SI Appendix*, Table S8) of CO+OH oxidation (30–32), which would drive δ^13^C and δ^18^O in opposite directions (black arrows). Instead, the observed trends likely reflected the influence of multiple interacting processes, including emissions, mixing, and sinks. In multisource systems, isotope mass balance approaches, such as the Keeling plot analysis, typically identify the dominant source contributing to elevated CO levels (*SI Appendix*, section S1). In our case, it represents the regional outflow from the IGP and broader South Asia. Keeling plot-derived source signatures (Fig. 2*B* and *SI Appendix*, Table S1) were remarkably consistent across years. They also show an increase in δ^13^C and a decrease in δ^18^O from BCOB (δ^13^C = −25.8 to −26.3‰, δ^18^O = 18.7 to 20.7‰) to MCOH (δ^13^C = −25.0 to −26.1‰, δ^18^O = 12.4 to 16.4‰). These spatial differences in derived source isotopic values are critical for understanding the atmospheric processing of CO in South Asia.

The isotopic evolution from BCOB to MCOH can be described by two major components: i) isotopic fractionation due to CO sinks (sink effect) and ii) the mixing of transported source CO, local background CO, and local secondary CO (mixing effect). Examples of SAPOEX2019 (Fig. 2*C*; South Asian Pollution Experiment 2019) and SAPOEX2024 (Fig. 2*D*) illustrate this interplay: blue markers denote Keeling-derived source signatures at BCOB and MCOH, black arrows and dashed lines represent the sink effect, while blue arrows and dashed lines indicate the mixing effect. At MCOH, the isotopic values of Keeling-derived sources fall along the fitted δ^13^C–δ^18^O lines (mixing effect), consistent with inputs from IGP source-region outflow that subsequently combine with local background air, local secondary CO, and remote marine air. This mixing effect is nearly orthogonal to the sink effect.

We use a discrete model calculation to illustrate the cumulative effects of mixing and sinks during transport. Transport from the IGP to the Maldives does not occur as an instantaneous 2,000 to 3,000 km jump. Instead, we consider hypothetical observation points spaced every 100 km along the transport pathway. The CO signal identified by the Keeling plot at the first site can be approximated as CO directly transported from the source region. The CO signal at the second site represents a mixture of aged source CO, local background CO at the first site, and secondary CO formed at the first site. This mixed air mass becomes the dominant upstream input to the region of the second site and therefore constitutes the signal identified by the Keeling plot at the second site. From this follows by analogy that the signal observed at the final site (MCOH) reflects long-term aged source CO, local background CO at the previous sites (new and aged), and secondary CO formed at the previous sites (new and aged). This final signal arriving at the MCOH receptor site thus represents an accumulated product of progressive aging and successive mixing along the transport pathway, rather than the original IGP source signature. Vector decomposition of these shifts quantifies the mean sink effect (black arrow in Fig. 2*C*) as +1.8 ± 0.1‰ for δ^13^C and –2.8 ± 0.2‰ for δ^18^O. While the Keeling-derived source at BCOB can be directly applied to IGP source apportionment, evaluating CO sources at MCOH requires correcting for the sink effect while retaining the mixing effect.

Long-range over-ocean transport from BCOB to MCOH hence represents a continuum from source-region outflow to increasingly diluted and mixed air masses (Fig. 2*E*). At BCOB, fresh CO emissions from combustion and oxidation of CH_4_ and NMVOCs dominate and the sink effect is comparatively small. Subsequent southward transport reduces CO levels and introduces isotopic shifts due to both OH-driven fractionation and mixing (sink and mixing effect). By the time air masses reach MCOH, they reflect extensive mixing of long-term aged source CO, background CO along the route, and secondary CO along the route. Further downstream over the remote Indian Ocean, continued aging further enhances sink-driven fractionation, but once concentration gradients diminish and local sources and sinks balance each other, the sink effect stabilizes. Overall, the source isotopic signatures at the two South Asian sites reflect the combined influence of sink and mixing effects. The sink effect needs to be corrected for, while the mixing effect accounts for the differences in source isotopic values between the two locations.

### 1.3. CO Source Information and Isotopic Source Signatures.

**Fig. 3.**
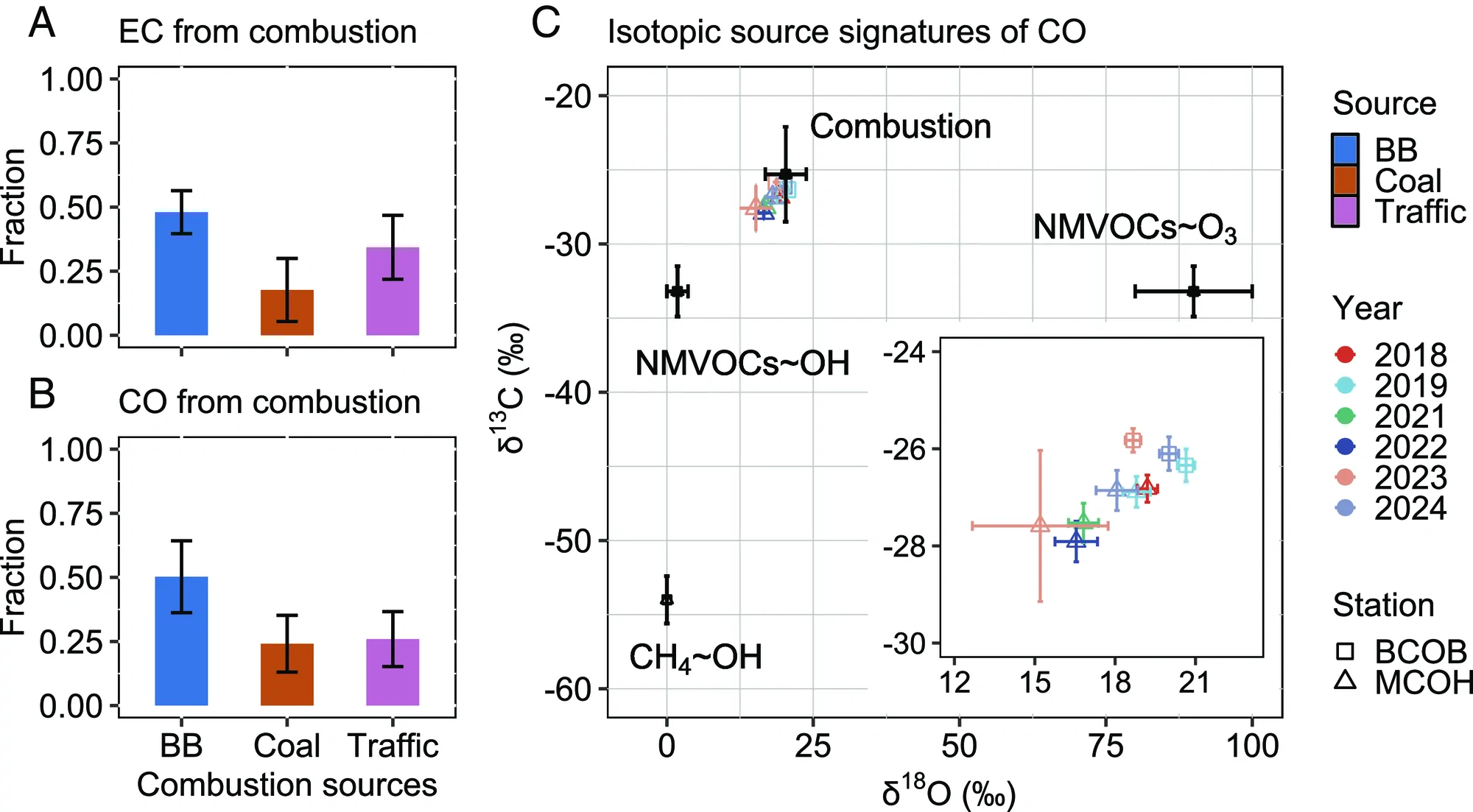
CO source information and isotopic source signatures. (*A*) Elemental carbon (EC) contributions from combustion sources, including biomass burning (BB), coal combustion, and traffic emissions, resolved via dual-isotope (δ^13^C and Δ^14^C) source apportionment (33). (*B*) CO contributions from combustion sources, calculated using CO/EC emission ratios (*SI Appendix*, Table S2 and Dataset S2) and EC fractions. (*C*) Keeling plot-derived source isotopic compositions (hollow squares and triangles, sink-corrected) and isotopic source signatures of CO (black markers). Isotopic source signatures of combustion sources were summarized from the literature and compiled into composite values by weighting each signature with its CO fraction using a Monte Carlo approach (*SI Appendix*, Table S2 and section S3.1). Isotopic source signatures of atmospheric oxidation-derived CO were summarized from the literature and further assessed with isotopic fractionation and atmospheric processes (*SI Appendix*, Tables S4 and S7).

After deducing the overall source and atmospheric processes reflected in the CO isotopes, we now turn to quantifying the relative contributions of specific sources. Combustion is regarded as the most important source of CO and has been extensively studied, providing a wealth of reference information. We therefore begin our analysis with combustion sources. Combustion processes coemit pollutants such as CO and elemental carbon (EC), which thus exhibit related emission patterns with fuel-specific CO/EC ratios being lower for traffic than for stationary combustion sources. Leveraging literature-based CO/EC emission ratios (*SI Appendix*, Table S2 and Dataset S2) and building on prior isotope-based EC source apportionment in South Asia (33) (Fig. 3*A*), we estimated CO source contributions using Monte Carlo simulations (Fig. 3*B* and Section 3.3). Biomass burning emerged as the dominant CO combustion source (50 ± 14%), followed by traffic emissions (26 ± 11%) and coal combustion (24 ± 11%). Recent dual-isotope studies of EC consistently indicate a high biomass burning contribution across many populated areas (20, 21), such as South Asia (33). This is based on higher CO/EC emission ratios for coal combustion and biomass burning than for traffic emissions (*SI Appendix*, Table S2 and Dataset S2). Traffic may be a key CO emission source in urbanized areas, while the contribution of biomass burning to CO for the full South Asian region may be underestimated.

After quantifying the relative contributions within combustion sources, we constructed region-specific combustion endmembers by integrating the fractional contributions of combustion sources (Fig. 3*B*) with corresponding isotopic values (Section 3.3 and *SI Appendix*, Table S2 and Dataset S1), in order to better constrain other CO sources. The resulting composite endmembers (Fig. 3*C*) provided a robust end-member reference database for source apportionment. Keeling plot-derived source values at BCOB (representing IGP outflow) and MCOH (representing the broader South Asian outflow; sink-corrected) clustered around the combustion endmembers, confirming combustion as the dominant regional CO source.

Beyond combustion, CO is also produced via secondary oxidation of CH_4_ and NMVOCs. Previous studies estimated that O_3_ oxidation of terpenes (a major NMVOC class) alone contributed to ~173 Tg y^−1^ CO globally (34), and kinetic data show that OH and O_3_ have comparable reactivity with many biogenic unsaturated NMVOCs (35, 36), indicating that both oxidants are important. We distinguish OH- and O_3_-driven oxidation based on their δ^18^O isotopic fingerprints (31, 37, 38) (*SI Appendix*, Table S4). OH-initiated pathways involve H-abstraction and subsequent incorporation of O_2_-derived oxygen (39), typically yielding moderate δ^18^O enrichment. In contrast, O_3_ contributes highly enriched δ^18^O directly (40). Minor oxidants such as Cl (41) and O(^1^D) (42) primarily shift δ^13^C without significantly affecting δ^18^O relative to the main sink, OH. Based on these distinctions, we defined three isotopically distinct oxidation-driven CO categories (Fig. 3*C* and *SI Appendix*, Tables S4 and S7). By establishing the above isotopic endmembers for combustion and atmospheric oxidation sources, we enable further isotopic source apportionment in South Asia and gain deeper insight into regional atmospheric oxidation processes.

### 1.4. Dual-Isotope Source Apportionment of CO.

**Fig. 4.**
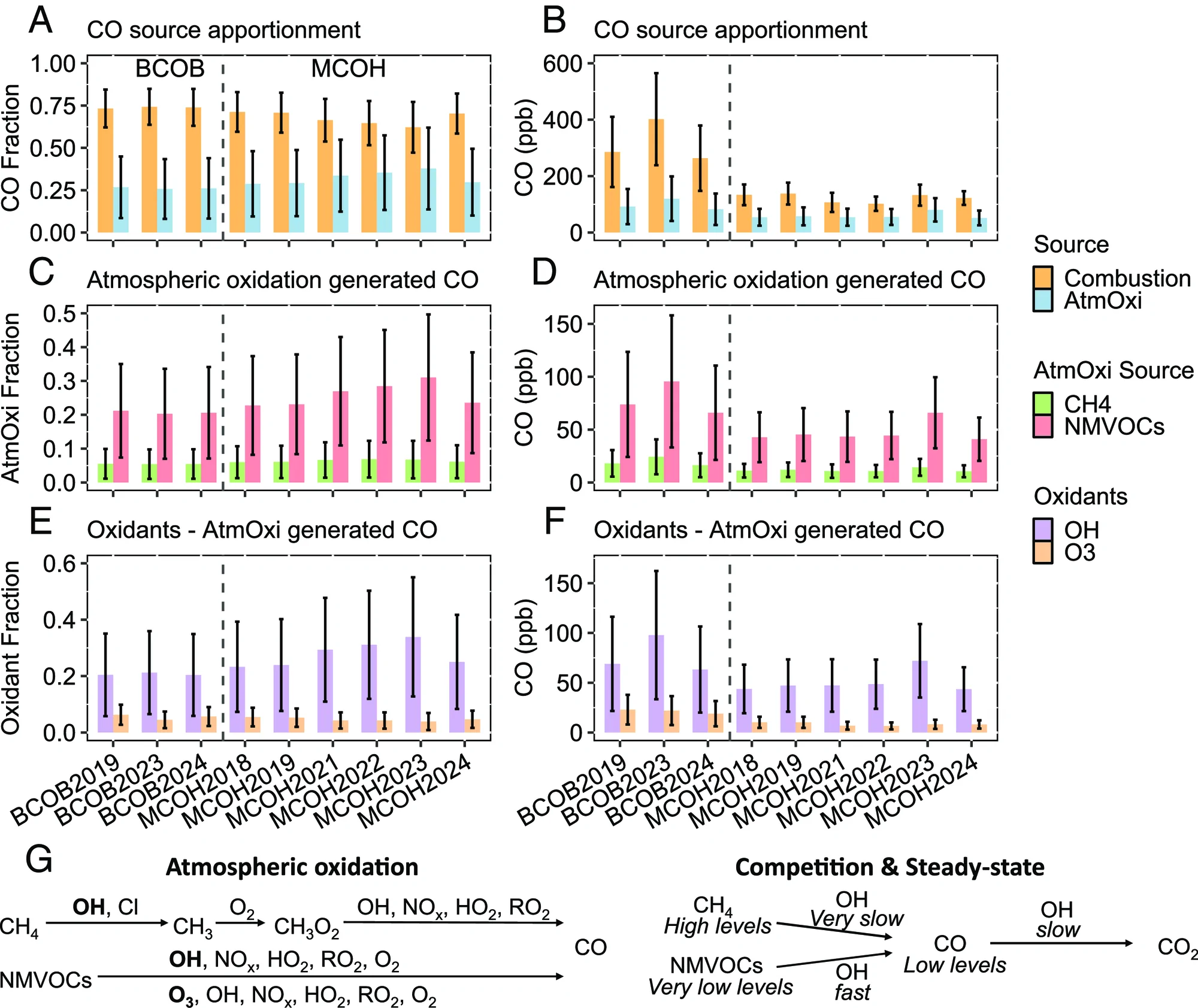
Multiyear dual-isotope source apportionment of CO at BCOB and MCOH: combustion, atmospheric oxidation pathways, and oxidant dynamics. (*A* and *B*) Source-resolved CO contributions: primary and secondary CO. (*C* and *D*) Precursor-specific oxidation pathways: CH_4_ and NMVOCs. (*E* and *F*) Oxidant regime analysis: OH-driven and O_3_-driven CO production. (*G*) Reaction pathways of atmospheric oxidation in CO source–sink dynamics, illustrating OH-driven and O_3_-driven pathways of CO production and the competition between CH_4_, NMVOCs, and CO for OH-driven oxidation.

To quantify CO sources in South Asia, we applied the MCMC model incorporating mass and dual-isotope constraints (*SI Appendix*, section S2). The model estimates the probability distribution of source contributions by exploring the endmember space that is consistent with the observations. Using Keeling plot-derived isotopic values, we source-apportioned CO at the IGP outflow site BCOB and the South Asian receptor site MCOH from 2018–2024 (Fig. 4 and *SI Appendix*, Tables S10–S11). Combustion sources dominated at both sites, contributing 74 ± 11% at BCOB and 68 ± 13% at MCOH (Fig. 4*A*). The contributions of atmospheric oxidation-derived CO are thus 26 ± 11% at BCOB and 32 ± 13% at MCOH, respectively, which is unexpectedly large for South Asian CO. Inventory-based models suggest that CO fluxes at BCOB are dominated by combustion (87%) with a minor atmospheric oxidation contribution (13%), whereas at MCOH the modeled contributions shift sharply to 12% from combustion and 88% from atmospheric oxidation (based on CO fluxes extracted from three models, *SI Appendix*, Table S12) (13–15, 17). However, the comparability between the model-derived source partitioning and our isotope-based source apportionment differs between the two sites.

At BCOB, located within the South Asian source region, intercepting the outflow from the IGP, a direct comparison between the two approaches is conceptually valid because both primarily reflect the regional source contribution pattern. Although inversion models may exhibit vertical variations in CO concentrations, such variations are expected to have a much smaller influence on their estimates of relative source fractions. This interpretation is supported by our observations at MCOH, where combustion-derived CO transported from South Asia still dominates the atmospheric signal despite the site being located more than 2,000 km from the source region. It is therefore reasonable to infer that the vertical gradients over only a few kilometers within the source region would still retain a similar regional source contribution pattern. Our isotope-based constraints therefore suggest that inventory-based models may overestimate the relative contribution of combustion sources in the South Asian source region. Notably, the isotope-based atmospheric oxidation contribution at BCOB (~26%) is approximately twice that derived from inventory-based models (~13%, *SI Appendix*, Table S12), indicating a significantly stronger-than-expected influence of atmospheric oxidation on CO over South Asia.

At MCOH, however, the comparison is less direct because the two approaches represent different aspects of the regional CO budget. The inversion models primarily describe regional CO production and flux partitioning within a transport-resolved framework, whereas the isotope-based constraints reflect the integrated atmospheric composition shaped by long-range transport and mixing. The persistence of a substantial combustion contribution at MCOH therefore indicates a strong influence of transported continental CO over the northern Indian Ocean. Rather than representing a contradiction, the two approaches provide complementary perspectives on regional CO sources and transport processes. Our isotope-based constraints thus offer an observational framework for evaluating transport-related uncertainties in current inversion models.

Secondary CO was dominated by NMVOC oxidation, which contributed 21% at BCOB and 26% at MCOH, while CH_4_ oxidation contributed only 5.5% and 6.4%, respectively (Fig. 4*C*). This resulted in a NMVOC:CH_4_ ratio of ~4:1, in contrast to model estimates (~2:1, *SI Appendix*, Table S12) (13). This suggested that existing models may underestimate the contribution of NMVOC oxidation to CO production in the South Asian atmosphere, which is heavily polluted not only by agricultural biomass burning but also by urban traffic emissions, industrial activities, and biogenic precursors emitted under high-temperature conditions (43, 44). These results underscore the significant, previously underestimated role of NMVOC oxidation in secondary CO production in South Asia.

Oxidant-specific contributions are rarely reported in models, and there are virtually no prior observations for validation. Our oxidant-specific partitioning showed that OH-driven oxidation accounted for 21 ± 11% of total CO at BCOB and 28 ± 14% at MCOH, while O_3_-driven oxidation contributed 5.5 ± 3.3% and 4.6 ± 3.1%, respectively (Fig. 4*E*). This yielded an OH:O_3_ ratio of ~4:1 for atmospheric oxidation-derived CO, indicating an essential role of O_3_, particularly in IGP source regions where its relative contribution is higher. Isotopic composition provided the quantitative observational constraints on O_3_-driven CO formation, filling a gap left by traditional models that lack direct observational validation.

Spatial patterns further emphasized that while the fractional contributions of CH_4_ and NMVOCs oxidation to secondary CO were similar at both sites (Fig. 4*C*), their absolute contributions were substantially higher at BCOB (Fig. 4*D*). This indicates enhanced oxidation of CH_4_ and NMVOCs in polluted regions, likely driven by OH regeneration and the involvement of O_3_ and NO_2_. Under such high-oxidant-demand conditions (Fig. 4*G*), NMVOCs likely outcompeted CO for oxidants, limiting CO removal.

Temporally, combustion-derived CO declined from 71 to 64% at MCOH during the COVID-19 pandemic, while oxidation-derived CO increased in both relative and absolute terms (Fig. 4 *A* and *B*). Although global OH levels were likely suppressed due to pollution and NO_2_ reductions, as suggested by model studies (45), the observationally based estimate of an increase in NMVOC-derived CO concentrations (Fig. 4*D*) indicated that reduced oxidative competition (from lower anthropogenic emissions) may have preferentially enhanced NMVOC oxidation. In contrast, CH_4_-derived CO concentrations remained stable, suggesting that reduced OH and reduced oxidative competition potentially cancel out in their impact on CH_4_ oxidation. During the same period, the contribution of O_3_-driven oxidation to total CO also declined from 5.4 to 4.1% at MCOH, while the OH-driven oxidation rose from 24 to 31%, reflecting the strong sensitivity of oxidant pathways to pollution levels (Fig. 4*F*).

These results demonstrated distinct sensitivities of CO, CH_4_, and NMVOCs to atmospheric oxidation. NMVOC oxidation was enhanced by both higher oxidant availability (pollution-enhanced oxidation) and reduced oxidative competition. In contrast, CO oxidation (i.e., removal) was largely suppressed under oxidative competition, with limited responsiveness to enhanced oxidants. The relatively stable contribution of CH_4_-derived CO indicates that the slow oxidation of CH_4_ is insensitive to short-term changes in oxidant abundance and competition. Together, these interactions define a steady-state oxidation regime (Fig. 4*G*) with important implications for comitigation strategies.

## 2. Discussion

### 2.1. Enhanced Atmospheric Oxidation.

**Fig. 5.**
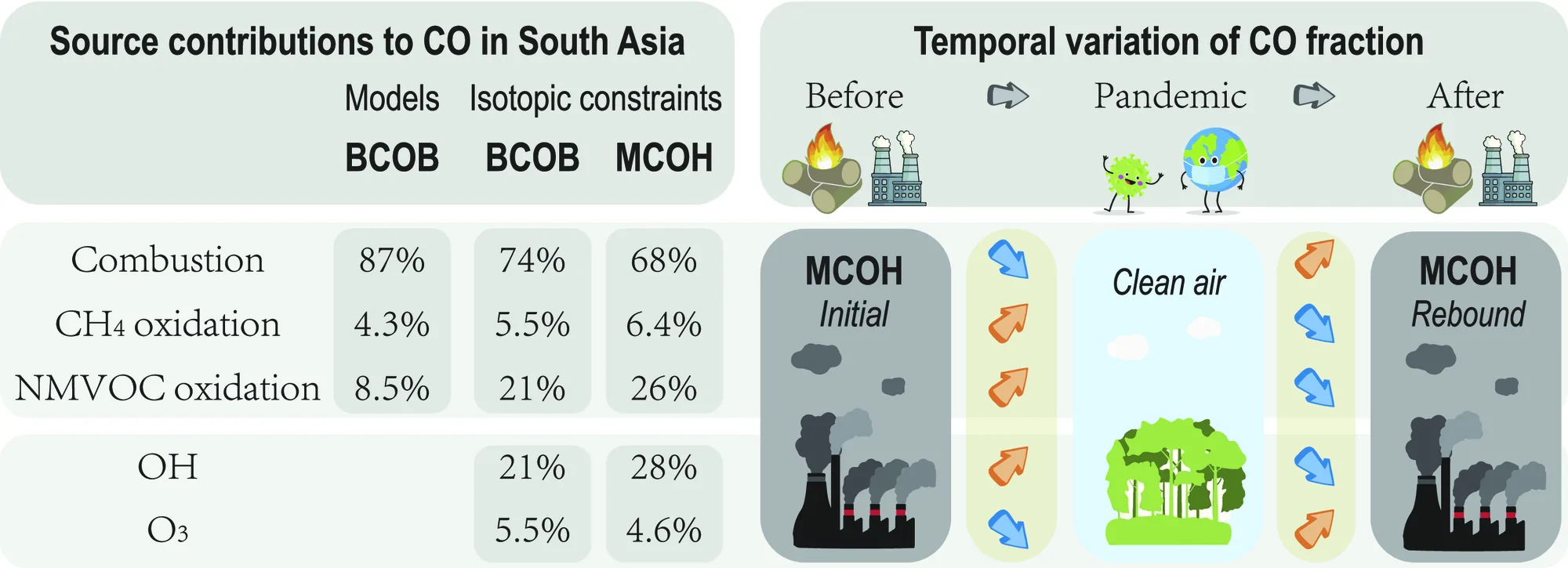
Spatial and temporal variations in CO source contributions in South Asia. (*Left*) Temporally averaged source contributions at BCOB (receptor for the IGP) and MCOH (broader South Asian receptor over the Indian Ocean) based on multiyear observations, with model estimates over South Asia included for comparison (*SI Appendix*, Table S12) (13–15, 17). (*Right*) Schematic illustration of temporal changes in CO sources at MCOH before, during, and after the COVID-19 pandemic (SAPOEX2018–2019, SAPOEX2020–2021, and SAPOEX2023–2024). The blue and yellow arrows denote the trends in the relative contributions of CO sources and the oxidants responsible for secondary CO production. Note that BCOB is not shown due to the lack of observations during the COVID-19 period. The CO sources include combustion, CH_4_ oxidation, and NMVOC oxidation. The oxidation of CH_4_ and NMVOC is mainly driven by OH and O_3_, leading to the formation of secondary CO.

The dual-isotope source apportionment presented here offers an independent observational constraint that bridges gaps between emission inventories, models, and inverse measurements of mixing ratios. Our results indicate that i) combustion sources dominated in the IGP source region but are lower than fractions estimated in models (13–15, 17), while secondary sources were of secondary importance yet exceed model-estimated contributions (Fig. 5); ii) over marine regions adjacent to the continent, CO remains primarily controlled by long-range transport from continental source regions, with local production contributing only a minor fraction; and iii) during the COVID-19 pandemic, combustion emissions decreased, whereas both the proportion and absolute levels of secondary CO sources increased, indicating weakened oxidative competition and enhanced removal of SLCFs.

Why could the fractional contribution of combustion sources be overestimated in inventories? Likely, total combustion emissions are generally better represented in emission inventories than secondary CO production, potentially arising from i) underestimated emissions and oxidation pathways of precursors such as NMVOCs, ii) underestimated OH recycling rates, and iii) overlooked contributions from other oxidants such as Cl, all of which warrant further investigation. Note that the IGP region has been found to exhibit an unusual chlorine enhancement, possibly from open burning of waste plastics (46). Expanded isotopic source apportionment can help to better constrain CO sources and atmospheric transport, correct systemic model biases, and more effectively guide mitigation strategies.

The unexpectedly large NMVOC-derived CO fraction indicates substantial underestimation of NMVOC emissions and a stronger oxidative capacity over South Asia than previously recognized, implying shorter regional lifetimes for NMVOCs, CH_4_, and CO. Model-based estimates of atmospheric oxidation typically derive from either the difference between bottom–up inventories and satellite-constrained total CO, or from chemical model constraints on OH levels (9–15, 47), precursor concentrations, and reaction rates, each carrying substantial uncertainties. In contrast, our isotope-based source apportionment directly constrains the atmospheric oxidation outcome, offering an independent benchmark. Major emission inventories (48–50) focus on anthropogenic sources, as natural emissions are difficult to quantify. Given that CH_4_ emissions and sinks are relatively well constrained (51), the ratio between CH_4_ and NMVOC oxidation-derived CO offers a pathway to better constrain global NMVOC budgets, particularly natural sources, thereby improving emission estimates and advancing understanding of climate–chemistry interactions.

### 2.2. Implications of Emission Mitigation for Air Quality and Climate Cobenefits.

The COVID-19 lockdown provided a natural experiment to evaluate atmospheric responses to emission reductions. Our isotope observations show that the decrease in primary CO emissions during this period exceeded the increase in secondary CO formation, resulting in a substantial reduction in regional CO burden despite concurrent changes in atmospheric oxidation chemistry. Although the relative contribution of methane oxidation increased during the lockdown, this primarily reflected the larger decline in primary CO sources rather than enhanced secondary CO production, consistent with the long atmospheric lifetime of CH_4_. In addition, reductions in overall anthropogenic emissions may enhance NMVOC removal by alleviating oxidative competition among reactive species (Fig. 5). These findings highlight the dominant role of emission reductions in controlling regional CO levels and improving air quality.

In contrast to declining global CO levels since 2000, anthropogenic CO emissions in South Asia have continued to rise (13, 52, 53), sustaining high regional burdens. As emission controls are strengthened in the future, atmospheric oxidation capacity may also respond to changes in CO, NMVOCs, NO_x_, and other reactive species. Reduced emissions may alter local OH recycling and thereby affect the removal of CO, NMVOCs, and CH_4_. However, our observations indicate that these chemical feedbacks are unlikely to offset the overall benefits of emission reductions. Coordinated mitigation strategies targeting CO, NMVOCs, CH_4_, and other ozone precursors therefore provide an effective pathway to achieve both climate and public-health benefits in heavily polluted regions. Expanding isotope observations across major source and receptor regions will further improve our ability to quantify emission-driven changes in atmospheric chemistry and evaluate future mitigation strategies.

## 3. Method

### 3.1. Ambient Air Sampling.

Air samples were collected at two receptor locations in South Asia. The Bangladesh Climate Observatory at Bhola Island (BCOB; 22.17° N, 90.71° E; 10 m agl) is located to intercept the integrated outflow from the IGP, approximately 300 km south of Dhaka, Bangladesh’s capital. Samples were taken at BCOB from November to May during winters 2019–2024, except for the COVID-19 pandemic winters of 2020–2022, when sampling was limited due to personnel and technical challenges. Synchronously, air samples were also collected at the Maldives Climate Observatory at Hanimaadhoo (MCOH; 6.78° N, 73.18° E; 15 m agl), strategically situated to intercept the large-footprint outflow from the greater South Asian region through its positioning on a northern island of the northernmost atoll in the Republic of Maldives, within the northern Indian Ocean. Samples were taken at MCOH from November to May winters 2018–2024 (except the COVID-19 pandemic winter of 2020 due to personnel and technical challenges). The campaigns were designated as part of the continuing South Asian Pollution Experiment, followed by the year, e.g., SAPOEX-2024 (covering winter from late 2023 to early 2024). During the winter sampling period, atmospheric circulation over South Asia is dominated by the northeast monsoon. Prevailing low-level northeasterly winds transport air masses from the IGP toward the Bay of Bengal and the northern Indian Ocean, facilitating strong continental outflow. As a result, BCOB intercepts relatively fresh emissions, integrating the IGP, whereas MCOH samples more aged air masses after long-range transport. All samples were collected during daytime. At each site, approximately 30 to 40 samples were collected per season. In some years, duplicate samples were obtained, whereas in others only single samples were collected. The typical sampling interval was approximately 3 d, although periods of both higher-frequency and more sparse sampling occurred. Detailed sampling information is provided in Dataset S3.

Cylindrical borosilicate glass flasks (1,000 mL) sealed at each end with a glass spindle valve with polychlorotrifluoroethylene cap (Normag, Germany) were used to collect and store air samples. Before shipment from Stockholm University, the flasks underwent a series of conditioning steps: vacuum (12 h), flushing with nitrogen evaporated from a liquid nitrogen tank (2 h), and a second evacuation step (3 h), before being flushed (1 h) and filled to 1.3 bar absolute pressure with synthetic air 5.0 (Strandmøllen, 20.9% oxygen, and 79.180% nitrogen, C_n_H_m_ ≤ 3 ppm, CO_2_ ≤ 1 ppm, CO ≤ 1 ppm, H_2_O ≤ 3 ppm). All conditioning steps were performed at 50 °C (26, 54). Portable flask samplers, equipped with a microdiaphragm pump (KNF Neuberger N86), were deployed at the two sites and connected to air inlets linked to 10 or 15 m tall observatory towers. The sampled air was first dried by passing through a stainless-steel trap containing anhydrous magnesium perchlorate (CAS# 10034−81−8, Alfa Aesar), secured with glass wool on both ends, followed by a 2 μm stainless steel particulate filter (Swagelok, Salon, OH) before entering the samplers. Following a 20-min flushing period, air samples were collected at a flow rate of 2 L min^−1^ to an absolute pressure of approximately 1.7 bar. A comprehensive dataset spanning three winters at BCOB (157 samples) and six winters at MCOH (286 samples) was thus collected, with some samples in duplicate for quality control.

CO surface mixing ratios were used from multispectral satellite data from NASA Earthdata, Giovanni, MOPITT, MOP03JM v009 (https://giovanni.gsfc.nasa.gov/giovanni). The CO mixing ratios were compiled for the period from November to April, spanning 2018–2024. Additionally, mixing ratios along the longitude of the BCOB and MCOH observatories were extracted for the respective winter. Additional atmospheric data for South Asia during winter from 2018–2024 were obtained and presented in *SI Appendix*, Figs. S11–S15, including ground-based PM_2.5_, satellite-derived surface CO, aerosol optical depth, total column ozone, and formaldehyde (HCHO) column density.

### 3.2. Isotopic Analysis of CO.

The analysis of CO mixing ratios and isotopic composition of the collected air samples was performed at the Institute for Marine and Atmospheric Research Utrecht (IMAU; Utrecht University) using continuous-flow isotope ratio mass spectrometry (IRMS, Thermo Scientific Delta V Advantage) (55). Air samples collected in glass flasks underwent CO extraction, with removal of CO_2_ and N_2_O facilitated by Ascarite/Mg(ClO_4_)_2_ and a liquid nitrogen trap. Subsequently, CO was oxidized to CO_2_ using Schütze reagent (sulfuric acid combined with I_2_O_5_ on granular silica gel) (55, 56), separated from O_2_ and N_2_ with a liquid nitrogen trap, purified with a gas chromatographic column, dried with a Nafion dryer, and finally transferred to the IRMS via an open split interface. The CO isotope values, corrected for the additional oxygen atom from the Schütze reagent, were derived from the CO_2_ isotope values (54, 55). Mixing ratios were determined by comparing peak areas of samples and standard gases and reported in parts per billion (ppb).

The isotope values are reported in δ notation, representing the relative deviation of the isotope abundance in a sample relative to international standards, i.e., Vienna Pee Dee Belemnite (V-PDB) for δ^13^C and Vienna Standard Mean Ocean Water (V-SMOW) for δ^18^O. Mixing ratios and isotope values underwent calibration using a reference gas of atmospheric air (NAT-307; CO = 180 ppb; δ^13^C= −30.25‰; δ^18^O = +7.10‰) (55). The precision (1 σ) for the measurements was estimated at 0.12‰ for δ^13^C and 0.16‰ for δ^18^O.

### 3.3. Dual-Isotope Source Apportionment.

To determine the isotopic values of the sources, we analyzed the isotopic results for each winter using both the Keeling plot (57) and Miller-Tans (58) methods (*SI Appendix*, Figs. S1–S9 and Table S1). The Keeling plot and Miller-Tans plot follow Eqs. **1** and **2**, respectively.

[1] $$\delta^{13}C_{\mathrm{obs}}=c_{\mathrm{bg}}\times \left(\delta^{13}C_{\mathrm{bg}}-\delta^{13}C_{\mathrm{source}}\right)\times \frac{1}{c_{\mathrm{obs}}}+\delta^{13}C_{\mathrm{source}},$$

[2] $$\delta^{13}C_{\mathrm{obs}}\times c_{\mathrm{obs}}-\delta^{13}C_{\mathrm{bg}}\times c_{\mathrm{bg}}=\delta^{13}C_{\mathrm{source}}\times \left(c_{\mathrm{obs}}-c_{\mathrm{bg}}\right),$$

Where *c* refers to the mixing ratio of CO, *obs* refers to atmospheric observation, *bg* refers to background, and *source* refers to source.

The isotopic source apportionment constraints are primarily based on mass conservation and isotopic conservation. Below are the mass balance equations for the source apportionment model.

[3] $$1=f_{\mathrm{Comb}}+f_{NMVOCs-OH}+f_{NMVOCs-O3}+f_{CH4-OH},$$

[4] $$\begin{array}{c} \delta^{13}C_{\mathrm{CO}}=\begin{array}{c} \;f_{\mathrm{Comb}}\times \delta^{13}C_{\mathrm{Comb}}+f_{NMVOCs-OH}\times \delta^{13}C_{NMVOCs-OH}\\ +f_{NMVOCs-O3}\times \delta^{13}C_{NMVOCs-O3}+f_{CH4-OH}\times \delta^{13}C_{CH4-OH} \end{array}, \end{array}$$

[5] $$\begin{array}{c} \delta^{18}O_{\mathrm{CO}}=\begin{array}{c} \;f_{\mathrm{Comb}}\times \delta^{18}O_{\mathrm{Comb}}+f_{NMVOCs-OH}\times \delta^{18}O_{NMVOCs-OH}\\ +f_{NMVOCs-O3}\times \delta^{18}O_{NMVOCs-O3}+f_{CH4-OH}\times \delta^{18}O_{CH4-OH} \end{array}, \end{array}$$

where *f* represents the fraction. *Comb* designates CO from combustion sources. *NMVOCs-OH* denotes CO from NMVOCs oxidized by OH. *NMVOCs-O_3_* represents CO from NMVOCs oxidized by O_3_. *CH_4_-OH* indicates CO from CH_4_ oxidized by OH. Eqs. **3**–**5** represent mass and isotopic mass balances. A MCMC model was developed for isotopic source apportionment of CO, and the mathematical algorithm is described in *SI Appendix*, section S2.

Sources were classified and merged based on the isotopic range and available information. Isotopic source signatures of CO from combustion were summarized from the literature (*SI Appendix*, Table S2 and Dataset S1), including biomass burning (δ^13^C = −26.6 ± 3.4‰ for C3 biomass, δ^13^C = −13.2 ± 2.4‰ for C4 biomass, δ^18^O = 18.2 ± 5.5‰), coal combustion (δ^13^C = −24.5 ± 2.9‰, δ^18^O = 22.0 ± 4.5‰), and traffic emissions (δ^13^C = −26.5 ± 9.8‰, δ^18^O = 22.6 ± 7.2‰). Fractions of each combustion source of elemental carbon (EC) were obtained based on isotopic source apportionment of EC in South Asia (33). The CO/EC ratios from combustion sources were collated from the literature (Dataset S2, summarized in *SI Appendix*, Table S2). Since both EC and CO are products of incomplete combustion, fractions of each combustion source of CO can be determined based on the isotopic source apportionment of EC and the CO/EC ratios of the combustion sources. The Monte Carlo method was used to propagate uncertainty. With the fractions of CO from different combustion sources and correlated isotopic source signatures, combustion sources were integrated into a single category (δ^13^C = − 25.3 ± 3.2‰, δ^18^O = 20.3 ± 3.5‰, *SI Appendix*, Table S2).

For secondary CO produced by atmospheric oxidation of CH_4_ and NMVOCs, isotopic source signatures from the literature were summarized in *SI Appendix*, Table S3. For the δ^18^O signatures of oxidation-derived CO, we adopted the widely accepted values, as listed in *SI Appendix*, Table S4. The determination of δ^13^C source signatures involved the original isotopic signatures of CH_4_ and NMVOCs (*SI Appendix*, Table S3), the proportions of OH and Cl radicals (*SI Appendix*, Table S5), and isotopic fractionation effects (*SI Appendix*, Table S6). By combining these factors, we calculated the δ^13^C isotopic signatures of CO produced from the oxidation of CH_4_ and NMVOCs as −54.0 ± 1.6‰ and −33.2 ± 1.7‰, respectively (*SI Appendix*, Table S7). Uncertainties were assessed using the Monte Carlo method. Detailed information is provided in *SI Appendix*, section S3. Sensitivity analyses of sink corrections and isotopic source signatures are presented in *SI Appendix*, Figs. S16–S18 and section S6.

## Supplementary Material

Appendix 01 (PDF)

Dataset S01 (XLSX)

Dataset S02 (XLSX)

Dataset S03 (XLSX)

## Data Availability

Isotopic composition of carbon monoxide in South Asia data has been deposited in Bolin Centre Database (10.17043/yao-2026-co-isotopes-1) (59). All other data are included in the article and/or supporting information.
