# Supplementary material for: Multiyear dual-isotope fingerprinting at South Asian receptor sites constrains carbon monoxide sources and enhanced oxidation
Source: Proc Natl Acad Sci U S A. 2026 Oct 12;123(42):e2606200123. doi: 10.1073/pnas.2606200123 (PMC13684142; doi:10.1073/pnas.2606200123)
Supplement: Supplementary file 1 [file pnas.2606200123.sapp.pdf]

Supporting Information

Multiyear dual-isotope fingerprinting at South Asian receptor sites constrains carbon monoxide sources and enhanced oxidation

Peng Yao[1]*, Henry Holmstrand[1], Carina van der Veen[2], Maria Elena Popa[2], Chloe A. Brashear[2], Krishnakant Budhavant[3,4], Mohanan Remani Manoj[1], Joakim Romson[1], Abdus Salam[5], Thomas Röckmann[2], Örjan Gustafsson[1]*

[1]Department of Environmental Science and Bolin Centre for Climate Research, Stockholm University, Stockholm 10691, Sweden.

[2]Institute for Marine and Atmospheric Research Utrecht (IMAU), Utrecht University, Utrecht 3584CC, The Netherlands.

[3]Maldives Climate Observatory-Hanimaadhoo (MCOH), Maldives Meteorological Services, H. Dh. Hanimaadhoo 02020, Maldives.

[4]Divecha Centre for Climate Change, Indian Institute of Science, Bangalore 560012, India.

[5]Department of Chemistry, University of Dhaka, Dhaka 1000, Bangladesh.

*Corresponding authors: Örjan Gustafsson (Orjan.Gustafsson@aces.su.se), Peng Yao (Peng.Yao@aces.su.se)

Table of contents

Sections S1–S6

Figs. S1–S18

Tables S1–S12

Data S1–S3

Data S1. Isotopic signatures of CO sources.

Data S2. Summary of combustion ratios.

Data S3. Raw data CO isotopes in South Asia.

References 1–40

S1. Isotopic source analysis of CO in South Asia

Isotopic source analysis of CO in South Asia over multiple years was performed using the Keeling plot and Miller-Tans plot methods (Figs. S1–S9 and Table S1). The reported uncertainty corresponds to the uncertainty in the slope of the linear fit.

Table S1. Isotopic values of the well-mixed source derived from the Keeling plot and Miller-Tans plot analyses. Unit in ‰.

Year	Site	Keeling				Miller-Tans			
		$\delta^{13}C$	$\delta^{13}C$_unc	$\delta^{18}O$	$\delta^{18}O$_unc	$\delta^{13}C$	$\delta^{13}C$_unc	$\delta^{18}O$	$\delta^{18}O$_unc
2018	MCOH	−25.02	0.28	16.40	0.38	−24.84	0.25	16.47	0.36
2019	BCOB	−26.34	0.33	20.65	0.32	−26.71	0.27	20.88	0.24
2019	MCOH	−25.09	0.32	16.00	0.52	−25.05	0.31	16.47	0.54
2021	MCOH	−25.73	0.41	14.02	0.56	−25.11	0.42	14.99	0.60
2022	MCOH	−26.11	0.42	13.75	0.79	−26.29	0.41	13.29	0.75
2023	BCOB	−25.83	0.24	18.67	0.29	−26.13	0.28	18.92	0.23
2023	MCOH	−25.79	1.56	12.41	2.53	−25.75	1.25	12.56	2.02
2024	BCOB	−26.10	0.35	20.01	0.36	−26.07	0.32	19.77	0.26
2024	MCOH	−25.06	0.41	15.27	0.78	−25.30	0.43	14.80	0.79

The Keeling plot was originally designed to isolate isotopic signatures from a single-source system. However, real-world atmospheric observations typically involve multiple contributing sources. To explore how the Keeling plot behaves under these conditions, we performed Monte Carlo simulations that incorporated multiple CO sources (Fig. S10). The background CO was set at $\delta^{13}C = -28‰$, $\delta^{18}O = 10‰$, with a CO mixing ratio of 100 ppb. A regional well-mixed inflow source was defined with $\delta^{13}C = -25 \pm 1‰$, $\delta^{18}O = 15 \pm 1‰$, and 100 ± 50 ppb, representing air masses well-mixed within the source region and transported to receptor sites. A local biomass-burning source (C4 vegetation) was defined with $\delta^{13}C = -13 \pm 1‰$, $\delta^{18}O = 22 \pm 1‰$, and either 10 ± 5 ppb (weak) or 400 ± 200 ppb (strong), representing locally emitted CO that could bias the

application of the Keeling plot. All scenarios assumed synchronous mixing and lognormally distributed isotopic values.

In Scenario 1, where only the regional well-mixed source is present, the Keeling plot accurately retrieves its isotopic signature (Fig. S10A–B). In Scenario 2, where a weak local biomass burning source is added, the Keeling plot still reflects the regional well-mixed source, indicating that minor contributions or background variability do not substantially bias the result (Fig. S10C–D). In Scenario 3, with a strong local biomass burning source, the Keeling plot shifts toward the biomass burning signature (Fig. S10E–F), demonstrating that the inferred source is dominated by the component contributing the highest CO concentration.

These simulations confirm that, in multi-source systems, the Keeling plot reflects the isotopic signature of the dominant CO source by concentration. In our case, the receptor sites are located in downwind regions where multiple CO sources are already well mixed, making the Keeling approach appropriate for capturing regional-scale source signatures.

S2. Mathematical algorithm: Markov Chain Monte Carlo (MCMC) model

$$1 = f_{Comb} + f_{NMVOCs-OH} + f_{NMVOCs-O3} + f_{CH4-OH} \quad (3)$$

$$\delta^{13}C_{CO} = f_{Comb} \times \delta^{13}C_{Comb} + f_{NMVOCs-OH} \times \delta^{13}C_{NMVOCs-OH} + f_{NMVOCs-O3} \times \delta^{13}C_{NMVOCs-O3} + f_{CH4-OH} \times \delta^{13}C_{CH4-OH} \quad (4)$$

$$\delta^{18}O_{CO} = f_{Comb} \times \delta^{18}O_{Comb} + f_{NMVOCs-OH} \times \delta^{18}O_{NMVOCs-OH} + f_{NMVOCs-O3} \times \delta^{18}O_{NMVOCs-O3} + f_{CH4-OH} \times \delta^{18}O_{CH4-OH} \quad (5)$$

Input:

Isotopic_data: A dataset with n samples, each containing observed isotopic values and corresponding source information.

N: Number of MCMC iterations.

burn: Burn-in period (initial samples to discard).

thin: Thinning interval (to reduce autocorrelation).

h: Jump size parameter for the perturbation step.

Output:

RESULT: A matrix containing the estimated mean and standard deviation of the fractional contributions x, y, z, v for sources A, B, C, D after MCMC sampling.

Steps:

S2.1 Initialization

Define the initial fractional contributions for the sources:

$$x_1 = y_1 = z_1 = v_1 = \frac{1}{4}$$

Define the perturbation variables p, q, r, s initialized to 1:

$$p_1 = q_1 = r_1 = s_1 = 1$$

S2.2 Iterative Sampling

For each sample j from 1 to n in *Isotopic_data*:

Extract the means $M_{A,j}$, $M_{B,j}$, $M_{C,j}$, $M_{D,j}$ and standard deviations $\sigma_{A,j}$, $\sigma_{B,j}$, $\sigma_{C,j}$, $\sigma_{D,j}$ for sources A, B, C, D.

Similarly, extract the means $N_{A,j}$, $N_{B,j}$, $N_{C,j}$, $N_{D,j}$ and standard deviations $\tau_{A,j}$, $\tau_{B,j}$, $\tau_{C,j}$, $\tau_{D,j}$ for the second isotopic data.

S2.3 MCMC Iterations (for each *i* from 2 to *N*)

Perturbation Step:

Perturb the variables p_i, q_i, r_i, s_i using:

$$p_i = p_{i-1} \times e^{h \times \epsilon_p}$$

$$q_i = q_{i-1} \times e^{h \times \epsilon_q}$$

$$r_i = r_{i-1} \times e^{h \times \epsilon_r}$$

$$s_i = s_{i-1} \times e^{h \times \epsilon_s}$$

Where ϵ_p , ϵ_q, ϵ_r, ϵ_s ~ $N(0,1)$

Normalization Step:

Normalize the perturbed variables to get the fractional contributions:

$$Total = p_i + q_i + r_i + s_i$$

$$x_i = \frac{p_i}{Total}$$

$$y_i = \frac{q_i}{Total}$$

$$z_i = \frac{r_i}{Total}$$

$$v_i = \frac{s_i}{Total}$$

S2.4 Probability Calculation

Primary Contribution Probability P_1:

$$P_1 = e^{\sum \log(x_i, y_i, z_i, v_i) - (x_i + y_i + z_i + v_i)}$$

Mean and Standard Deviation for Isotopic Values:

$$\mu_M = x_i \times M_{A,j} + y_i \times M_{B,j} + z_i \times M_{C,j} + v_i \times M_{D,j}$$

$$\sigma_M = \sqrt{\left(x_i \times \sigma_{A,j}\right)^2 + \left(y_i \times \sigma_{B,j}\right)^2 + \left(z_i \times \sigma_{C,j}\right)^2 + \left(v_i \times \sigma_{D,j}\right)^2}$$

Similarly calculate μ_N and σ_N.

Secondary Contribution Probabilities P_2 and P_3:

$$P_2 = \frac{1}{\sqrt{2\pi{\sigma_M}^2}} \times e^{-\frac{(Observed_M - \mu_M)^2}{2{\sigma_M}^2}}$$

$$P_3 = \frac{1}{\sqrt{2\pi{\sigma_N}^2}} \times e^{-\frac{(Observed_N - \mu_N)^2}{2{\sigma_N}^2}}$$

Overall Probability *P[i]*:

$$P[i] = P_1 \times P_2 \times P_3$$

S2.5 Metropolis-Hastings Criterion

If

$$\frac{P[i]}{P[i-1]} < Uniform(0,1)$$

reject the new state:

$$x_i = x_{i-1}$$

$$y_i = y_{i-1}$$

$$z_i = z_{i-1}$$

$$v_i = v_{i-1}$$

Otherwise, accept the new state.

S2.6 Post-processing

Compute the acceptance rate.

Discard the burn-in period samples and apply thinning:

$$x_{final} = x[\text{burn} + 1{:}\, N{:}\, \text{thin}]$$

Calculate the mean and standard deviation of x, y, z, and v over the thinned samples.

S2.7 Store and Output Results

Append the results to the RESULTS matrix and save it to a file.

This algorithm captures the essence of the MCMC method, mathematically describing the iterative sampling, constraint enforcement, and probability-based acceptance mechanism used to estimate the source contributions under specific isotopic constraints.

S3. Isotopic source signatures of CO

S3.1 Combustion sources of CO

CO typically originates from inefficient combustion, with primary sources encompassing biomass burning, coal combustion, and traffic emissions. Isotopic signatures of CO from combustion sources were collated from the literature, as shown in Data S1 and summarized in Table S2.

Table S2. Compiled isotopic signatures of CO from combustion sources.

Calculation	Item	Biomass burning	Coal combustion	Traffic emissions	Data from
Isotopic source signatures (Literature review)	δ^{13}C of CO (‰)	C3: −26.6 ± 3.4<br>C4: −13.2 ± 2.4	**−24.5 ± 2.9**	**−26.5 ± 9.8**	Data S1
	δ^{18}O of CO (‰)	**18.2 ± 5.5**	**22.0 ± 4.5**	**22.6 ± 7.2**	Data S1
EC dual-isotope source apportionment (Observation)	EC fraction	C3: 0.426 ± 0.044<br>C4: 0.054 ± 0.040<br>**Combined: 0.480 ± 0.084**	**0.177 ± 0.123**	**0.343 ± 0.125**	South Asia
Literature review (Median, 95% CI)	CO/EC ratio	**175.7 (154.7–205.5)**	**247.8 (185.0–366.9)**	**136.1 (79.5–274.5)**	Data S2
CO fractions based on EC fractions and CO/EC ratios	CO fraction	C3: 0.444 ± 0.095<br>C4: 0.055 ± 0.030<br>**Combined: 0.502 ± 0.140**	**0.241 ± 0.111**	**0.259 ± 0.107**	EC-based
**MC combination**	**All combined**	**δ^{13}C = −25.3 ± 3.2‰**<br>**δ^{18}O = 20.3 ± 3.5‰**			

In CO from biomass burning, C3 and C4 plants are distinguished by their markedly different δ^{13}C source signatures. The δ^{13}C isotopic signatures were collected from the literature, as shown in Data S1 and summarized in Table S2. Globally, raw materials from C3 and C4 plants have δ^{13}C source signatures of −27.2 ± 1.7‰ and −13.2 ± 1.1‰, respectively (1). Consequently, the isotope fractionations (Δ^{13}C) of CO from biomass burning are minimal, roughly 0.6‰ for C3 biomass and 0.0‰ for C4 biomass. The direction of these isotope fractionations aligns with findings from a recent study (2).

For CO emissions from traffic, isotopic signatures are influenced by factors such as fuel type (natural gas, gasoline or diesel), analysis approach (individual vehicles or fleet averages), and the

geological origin of the crude oil used (1). While hundreds of source data points have been reported in the literature, the number of studies remains limited. Therefore, we rely on average values for evaluation rather than further stratifying the source data, as shown in Table S2.

For CO from coal combustion, there are very few studies that have reported $\delta^{13}C$ source signatures. However, based on the minimal isotope fractionation in biomass burning and traffic emissions, as well as findings from previous studies (1, 2), coal combustion is also expected to exhibit very small isotope fractionations. Using the global average $\delta^{13}C$ source signature of −24.5 ± 0.8‰ (1) and incorporating the uncertainty from a coal pyrolysis-generated CO study (3), we estimate the $\delta^{13}C$ source signature for CO from coal combustion to be −24.5 ± 2.9‰, as shown in Table S2.

Elemental carbon (EC) in atmospheric aerosols originates exclusively from combustion. Our previous study (4) provided $\delta^{13}C$ and $\Delta^{14}C$ data for EC in BCOB and MCOH. This uncertainty was derived from the probability density in the MCMC method. The results were then combined to represent both sites as proxies for South Asia.

We conducted a comprehensive review of ratios among combustion products, as shown in the file Data S2, where the CO/EC ratios were summarized for each combustion source and shown in Table S2. The ratios are dimensionless and calculated by comparing the emission factors of CO and EC, expressed in units of g kg^{-1} fuel or g km^{-1} $vehicle^{-1}$.

Combining the EC fractions from isotopic source apportionment in South Asia and CO/EC ratios, we achieved an EC-based source apportionment of CO from combustion sources in South Asia with the Monte Carlo method, as shown in Table S2. The specific operation was to multiply the corresponding EC fractions and CO/EC ratios, then normalize. Uncertainty was determined with Monte Carlo error propagation.

By combining isotopic source apportionment of EC in South Asia with summarized CO/EC ratios, we can compile the three major combustion sources into a single set of isotopic signatures for combustion emissions. By multiplying the CO fractions from combustion sources and corresponding isotopic source signatures, we finally determined a compiled isotopic source signature of CO for all combustion sources, i.e., $\delta^{13}C = -25.3 \pm 3.2‰$ and $\delta^{18}O = 20.3 \pm 3.5‰$ in South Asia, as shown in Table S2. To evaluate the impact of source variability on the $\delta^{13}C$ signatures of combustion emissions, we introduced a 10% perturbation in the combustion source proportions to simulate potential fluctuations in combustion emissions. The resulting changes in the $\delta^{13}C$ source values were minimal.

S3.2 Atmospheric oxidation-derived CO

S3.2.1 δ^{18}O source signature of CO from atmospheric oxidation

Table S3. Isotopic source signatures of CO from atmospheric oxidation in the literature.

Oxidant	Source	δ^{13}C (‰) V-PDB	δ^{18}O (‰) V-SMOW	Ref.
OH	NMVOCs	−32.2	15	(5)
OH	NMVOCs		0	(6), cited in (7)
OH/Cl	NMVOCs	−32.8 ± 0.5	3.6 ± 1.2	(8)
OH	CH_4	−52.6 (−47.2, Δ^{13}C = 5.4)		(9), cited in (10)
OH	CH_4	−51.1 (−47.1, Δ^{13}C = 4)		(11), cited in (12)
OH	CH_4	−55 (−47.8, Δ^{13}C = 7.2)		Ref. cited in (5)
OH	CH_4		14.9	(5)
OH	CH_4		0	(6), cited in (7)
OH	CH_4		39.1	(13)
O_3	NMVOCs		80–100	(14)
O_3	CH_4		80–100	(14)

The determination of this δ^{18}O source signature of CO from atmospheric oxidation primarily involves the mechanisms of oxidation of CH_4 and NMVOCs, as well as the sources of oxygen atoms. The OH-driven oxidation mechanisms of CH_4 and NMVOCs are analogous, with Cl and O(^{1}D) radicals also included. In the initial step, hydrogen is removed by OH radicals, forming CH_3 radicals. Subsequently, CH_3 radicals combine with O_2, as depicted in Equations S1–S4.

$$\mathrm{CH_4 + OH \rightarrow H_2O + CH_3} \quad (S1)$$

$$\mathrm{CH_4 + Cl \rightarrow HCl + CH_3} \quad (S2)$$

$$\mathrm{CH_4 + O(^1D) \rightarrow OH + CH_3} \quad (S3)$$

$$\mathrm{CH_3 + O_2 \rightarrow CH_3O_2} \quad (S4)$$

This initial step establishes that the oxygen involved in the oxidation process originates from O_2, extending to the formation of the oxidation product CO. Consequently, CH_3O_2 is expected to

exhibit a similar $\delta^{18}O$ source signature to that of atmospheric O_2. Subsequent oxidation processes may show selectivity towards specific CH_3O_2, but are unlikely to induce significant isotope fractionation in the resulting oxidation product, CO. The atmospheric oxygen is characterized by a $\delta^{18}O$ value of around 23.7‰ (average of 23.5‰ (13) and 23.9‰ (15)).

We compiled various $\delta^{18}O$ source signatures of CO from atmospheric oxidation sources from the literature, as presented in Table S3. There is debate over the $\delta^{18}O$ source signatures from the atmospheric oxidation route and relevant studies are scarce (5, 6, 13). We adopted the isotopic signatures commonly used in the field, as shown in Table S4, but determining which values are most accurate will require further in-depth research.

Table S4. $\delta^{18}O$ source signatures of CO from atmospheric oxidation.

Source	$\delta^{18}O$ (‰)	Ref.
CH_4 + OH/Cl	0	Ref in Table S3
NMVOCs + OH/Cl	1.8 ± 1.8	Ref in Table S3
NMVOCs + O_3	90 ± 10	(14)

S3.2.2 $\delta^{13}C$ source signatures of CO from atmospheric oxidation

Researchers have essentially reached a consensus regarding the $\delta^{13}C$ source signatures of CO resulting from atmospheric oxidation. However, these isotopic source signatures are influenced by both the carbon source and the isotope fractionation caused by various oxidants. Therefore, in this section, we introduced a hybrid approach to determine the range of $\delta^{13}C$ signatures of CO from atmospheric oxidation in specific regions.

While the literature generally agrees on the approximate range of the $\delta^{13}C$ source signature of CO from atmospheric oxidation, several challenges persist. Firstly, there is uncertainty about the ratio of CH_4 to NMVOC oxidation in the atmosphere, and secondly, the ratios of the oxidants involved. Therefore, we propose a method that considers both ratios to determine the $\delta^{13}C$ signature of CO from atmospheric oxidation in specific regions.

The $\delta^{13}C$ source signatures of CO from atmospheric oxidation are strongly influenced by the differing isotope fractionations of $\delta^{13}C$ during CH_4 and NMVOCs oxidation by OH and Cl radicals. The relative contributions of OH and Cl radicals to atmospheric oxidation have been summarized from the literature, drawing on various modeling approaches, as detailed in Table S5.

Table S5. Fractions of OH and Cl radicals participating in atmospheric oxidation in the literature.

Source	Location	Type	OH (%)	Cl (%)	Ref.
CH_4	Global		98.1	1.9	(16) IPCC
CH_4	Lower stratosphere		91	9	(17)
CH_4	Marine site			8.2	(18)
CH_4	Global troposphere			1.0	(19)
CH_4	Troposphere			2.6	(20)
NMVOCs	Marine site	C_2–C_8 alkanes		32.5 ± 11.8	(18)
NMVOCs	Marine site	C_2–C_8 unsaturated hydrocarbon		5.9 ± 0.6	(18)
NMVOCs	Global troposphere	Ethane		20	(19)
NMVOCs	Global troposphere	Propane		14	(19)
NMVOCs	Global troposphere	Methanol		4	(19)

As summarized in Table S5, literature reports indicated that the fraction of Cl radicals involved in the atmospheric oxidation of CH_4 ranged from 1% to 9%. However, a recent study suggested that the role of tropospheric Cl as a CH_4 sink was very limited, with its impact much smaller than previously estimated (21). Given the variability in Cl participation across different models, we used the mean value as an estimate. For NMVOCs, the fraction of Cl radicals in atmospheric oxidation ranged from 4% to 32.5%, reflecting significant and variable isotope fractionation. While determining the precise isotope fractionation for total NMVOCs is challenging, it can be reasonably estimated to fall within a specific range (22). Considering the similar reaction mechanisms involving hydrogen removal from carbon chains (as outlined in Equations S1–S4), we assumed that NMVOCs underwent oxidation processes analogous to those of CH_4.

Table S6. Reaction rate ratios of different isotopes in the CH_4 oxidation.

Oxidants	Condition	α_C	Ref.
OH	296 K	1.0039 (± 0.0004, 2 σ)	(11)
OH	273–353 K	1.0054 (± 0.0004, 2 σ)	(23)
OH	297 ± 3 K	1.010 (± 0.007, 2σ)	(24)
OH		1.003	(25)
Cl	297 K, N_2 and N_2/O_2	1.066	(26)
Cl	298 ± 2 K, 1013 ± 10 mbar	1.058 (± 0.002)	(27)
Cl	299 K	1.0621 (± 0.0001 2σ)	(28)
Cl	296–300 K	1.026 *	Tanaka et al., 1996, cited in (28)
Cl	296–300 K	1.013–1.020 *	Tanaka et al., 1997, cited in (28)
Cl	296–300 K	1.04 *	Gupta et al., 1997, cited in (28)
Cl	296–300 K	1.06	Roberto-Neto et al., 1998, cited in (28)
Cl	296–300 K	1.066	Crowley et al., 1999, cited in (28)
$O(^1D)$	223–295 K	1.013	(11)
Summary	CH_4 + OH	α_C = 1.0056 ± 0.0031	
	CH_4 + Cl	α_C = 1.0624 ± 0.0032	
	$CH_4 + O(^1D)$	α_C = 1.013	

Table S7. $\delta^{13}C$ source signature of CO from atmospheric oxidation of CH_4 and NMVOCs over continental and oceanic atmosphere in the South Asia-Indian Ocean region.

	CH_4	NMVOCs	Ref.
Raw materials $\delta^{13}C$	−47.5 ± 0.1‰ (29, 30)	−26.5 ± 1.2‰ (31, 32)	CH_4 (29, 30); NMVOCs (31, 32)
OH oxidation - $\Delta^{13}C$	−5.5 ± 1.6‰ (5, 9–12)	−5.7 ± 1.2‰ (5, 31, 32)	Table S3
OH oxidation - fraction	0.982 ± 0.008	0.982 ± 0.008	Table S5 (16, 19, 20)
Cl oxidation - $\Delta^{13}C$	−61.6‰ (11.2 times)	−63.8‰ (11.2 times)	Calculated from Table S6
Cl oxidation - fraction	0.018 ± 0.008	0.018 ± 0.008	Table S5 (16, 19, 20)
$O(^1D)$ oxidation - $\Delta^{13}C$	−12.8‰ (2.33 times)	−13.3‰ (2.33 times)	Calculated from Table S6
$O(^1D)$ oxidation - fraction	0	0	Negligible
Atmospheric oxidation $\delta^{13}C$	−54.0 ± 1.6‰	−33.2 ± 1.7‰	Weighted average of OH and Cl

The isotope fractionation of CH_4 + OH is, on average, $\Delta^{13}C$ = −5.5‰ in Table S3. Biogenic emissions (e.g., isoprene) were observed to have $\delta^{13}C$ values of −25.23 ± 1.44‰ in Southwest China (31) and −27.7 ± 2.0‰ from Velvet Bean (32), with an average of −26.5 ± 1.2‰. Compared with the $\delta^{13}C$ source signatures of NMVOCs (−32.2‰) (5), the isotope fractionation was also approximately $\Delta^{13}C$ = −5.7‰. Therefore, it is reasonable to take the estimation that $\delta^{13}C$ isotope fractionations of CH_4 and NMVOCs oxidation are similar in atmospheric oxidation.

The reaction rate ratios of CH_4 oxidation were compiled in Table S7. Based on the isotope fractionation of CH_4 + OH discussed above ($\Delta^{13}C$ = −5.5‰), additional isotope fractionations can be assessed by comparing α_C among OH radicals and various oxidants. As per the reaction rate ratios in Table S6, Cl and $O(^1D)$ radicals can induce 11.2- and 2.33-fold higher ^{13}C isotope fractionation, respectively, compared to the isotope fractionation in the CH_4 + OH reaction.

To derive the $\delta^{13}C$ source signatures of CO from the atmospheric oxidation of CH_4 and NMVOCs, detailed information was compiled in Table S7. The $\delta^{13}C$ source signature of atmospheric CH_4 was based on the recent global average (30), and for NMVOCs, it was determined using the mean isoprene (biogenic) value from relevant literature (31, 32). As discussed above, the isotope

fractionations ($\Delta^{13}C$) were established at −5.5‰ and −5.7‰ for OH oxidation of CH_4 and NMVOCs, respectively. Oceanic atmospheric oxidation oxidant participation fractions were sourced from global averages in the literature (16, 19, 20), with 0.982 for OH and 0.018 for Cl radicals. Considering similar reaction principles involving H removal from the carbon chain, we assumed equal participation in NMVOCs as in CH_4. Considering that combustion sources dominate the CO budget in South Asia, the influence of Cl participation on the isotopic composition of atmospheric CO is comparatively minor. We therefore fixed the contribution of Cl oxidation to reduce the number of free parameters and improve the constraints on the source apportionment. With these oxidant fractions, the isotope fractionations ($\Delta^{13}C$) for the atmospheric oxidation of CH_4 and NMVOCs were calculated to be −6.5‰ and −6.7‰, respectively. Consequently, the final $\delta^{13}C$ source signatures were −54.0 ± 1.6‰ and −33.2 ± 1.7‰ for the atmospheric oxidation of CH_4 and NMVOCs, respectively.

O_3-driven oxidation of NMVOCs is well characterized in atmospheric chemistry but remains poorly constrained isotopically, with no established $\delta^{13}C$ source signatures (14). In the absence of evidence for significant $\delta^{13}C$ fractionation differences between OH- and O_3-initiated oxidation, we applied $\delta^{13}C$ values from OH oxidation of NMVOCs as a proxy for O_3-derived CO.

Finally, a sensitivity test on the isotopic signatures of oxidation-derived CO sources showed minimal impact on overall source apportionment. This insensitivity arose from the large isotopic contrast between well-mixed atmospheric CO (as derived from Keeling plots) and the oxidation-derived endmember values.

S4. Atmospheric removal of CO

In previous studies, reaction rate ratios of different isotopes in the CO + OH reaction have been reported in α notation or as kinetic isotope fractionation effects (KIE) based on experiments or observations. In this context, we consolidated all the data into α notation to facilitate derivations and calculations, as depicted in Table S8.

Table S8. Reaction rate ratios of different isotopes in the CO + OH reaction.

	Condition	α_C	α_O	Ref.
CO + OH	1 atm, air; laboratory	1.007		(33)
	0.2–1 atm, air; laboratory		0.990	(33)
	Atmosphere; observatory		0.992	(7)
	Atmosphere; observatory	1.00579		(34) cited in (35)
	Atmosphere; observatory	1.011 (± 0.005)	0.985 (± 0.005)	(34) cited in (36)
	Arctic; observatory	1.0065		(37)
	Arctic; observatory		0.985	(37)
Overall	α	$\alpha_C = 1.0076 \pm 0.0023$	$\alpha_O = 0.9880 \pm 0.0036$	

Atmospheric conditions, such as pressure and temperature, can influence reaction rate ratios. As indicated by a previous study (33), α_O changes slightly with pressure variations from 0.2 to 1 atm, but α_C undergoes significant changes, ranging from 0.997 to 1.008, when the pressure changes from 0.2 to 1.4 atm (1.007 at 1 atm). However, since most atmospheric conditions experience only slight pressure variations, the summarized value in Table S8 is applicable to the majority of atmospheres.

S5. Atmospheric information during the campaign periods

Atmospheric information for South Asia during winter from 2018 to 2024 includes:

(a) Ground-based $PM_{2.5}$ concentrations in Dhaka from 2016 to 2024 (Fig. S11), offering insight into local air quality trends across the lockdown and reopening periods.

(b) Surface CO mixing ratios from MOPITT (Fig. S12), illustrating spatial patterns and interannual variability in near-surface CO levels.

(c) Aerosol optical depth (AOD) at 550 nm from MODIS (Fig. S13), capturing aerosol loading over land and ocean.

(d) Total column ozone from AIRS (Fig. S14), reflecting regional-scale oxidative capacity changes.

(e) Formaldehyde (HCHO) column density from OMI (Fig. S15), serving as a proxy for CH_4 and NMVOC oxidation activity.

S6. Details of isotopic source apportionment and literature-based models

S6.1 Isotopic source apportionment in South Asia

Table S9. Source apportionment of elemental carbon (EC) and carbon monoxide (CO) in South Asia.

Source	EC	ECunc	CO	COunc
BB	0.480	0.084	0.502	0.140
Coal	0.177	0.123	0.241	0.111
Traffic	0.343	0.125	0.259	0.107

Table S10. Dual-isotope source apportionment of CO in MCOH.

Site	Combustion	CH_4-OH	NMVOCs-OH	NMVOCs-O_3
MCOH2018	0.712 ± 0.117	0.060 ± 0.047	0.173 ± 0.113	0.055 ± 0.033
MCOH2019	0.708 ± 0.118	0.061 ± 0.048	0.178 ± 0.115	0.053 ± 0.032
MCOH2021	0.664 ± 0.126	0.066 ± 0.052	0.227 ± 0.131	0.043 ± 0.029
MCOH2022	0.647 ± 0.130	0.069 ± 0.054	0.242 ± 0.137	0.043 ± 0.029
MCOH2023	0.622 ± 0.150	0.068 ± 0.055	0.271 ± 0.156	0.039 ± 0.030
MCOH2024	0.703 ± 0.118	0.061 ± 0.049	0.189 ± 0.118	0.047 ± 0.031

Table S11. Dual-isotope source apportionment of CO in BCOB.

Site	Combustion	CH_4-OH	NMVOCs-OH	NMVOCs-O_3
BCOB2019	0.733 ± 0.112	0.055 ± 0.044	0.149 ± 0.103	0.063 ± 0.036
BCOB2023	0.743 ± 0.106	0.054 ± 0.044	0.158 ± 0.103	0.045 ± 0.029
BCOB2024	0.739 ± 0.109	0.055 ± 0.044	0.149 ± 0.101	0.057 ± 0.034

S6.2 Sensitivity tests

To assess the influence of isotopic fractionation during transport, we conducted a sensitivity analysis of source apportionment for BCOB and MCOH (Fig. S16). BCOB, being near major emission sources, was characterized by freshly emitted or recently formed CO with minimal sink influence. In contrast, CO at MCOH was subject to long-range transport and atmospheric aging, requiring correction for sink effects during transit from BCOB. We compared the source apportionment results at MCOH before and after applying the sink correction. Overall, the estimated combustion contribution differed by ~5%, indicating that while sink effects exert a measurable influence, they do not alter the overall temporal variability or inferred trends in source contributions.

Given the limited number of measurements constraining $\delta^{18}O$ signatures, we performed a sensitivity analysis to evaluate the influence of combustion and atmospheric oxidation (OH-driven) $\delta^{18}O$ endmembers on the source apportionment results (Figs. S17–S18). The mean $\delta^{18}O$ values for both combustion and atmospheric oxidation were perturbed by ±2‰, while their associated uncertainties were kept unchanged, to assess the impact on inferred source contributions and the robustness of the apportionment. The results show that these perturbations have only a minor effect on the apportionment outcomes. This limited sensitivity arises because the $\delta^{18}O$ signatures of different CO sources are well separated, such that a ±2‰ shift represents a relatively small perturbation in source space and therefore exerts only a modest influence on the inferred source contributions. Therefore, uncertainties in $\delta^{18}O$ signatures are unlikely to have a substantial impact on the overall conclusions of this study.

S6.3 Models in the Literature

Table S12. Source contributions from literature-based models focusing on South Asian winter seasons.

Model	Source	Combustion	Secondary	CH_4	NMVOCs
Zhe_2017(38)	South Asia	0.768	0.232	0.066	0.166
	BCOB	0.773	0.227	0.074	0.153
	MCOH	0.005	0.995	0.995	0.000
Zheng_2019(39)	South Asia	0.863	0.137	0.057	0.081
	BCOB	0.898	0.102	0.036	0.066
	MCOH	0.116	0.884	0.586	0.298
Nus_2025(40)	South Asia	0.905	0.095		
	BCOB	0.945	0.055		
	MCOH	0.227	0.773		
Summary	South Asia	0.845	0.155	0.052	0.103
	BCOB	0.872	0.128	0.043	0.085
	MCOH	0.116	0.884	0.744	0.140

Based on global data from the models (38–40), which provide the spatial distribution of various sources worldwide, we conducted a focused analysis for the South Asia region. Here, “South Asia” refers to the cumulative land area encompassing India, Bangladesh, Pakistan, Nepal, Bhutan, Sri Lanka, and the Maldives, excluding ocean areas. We calculated the source contributions within this defined region. Similarly, we calculated source contributions specifically for Bangladesh to represent the BCOB site. Since MCOH is located on the Maldives islands, whose area is too small to match the model’s latitude-longitude resolution, we instead selected a 5°×5° grid box centered around MCOH to estimate source contributions. For the datasets from Zhe et al. (2017) (38) and Zheng et al. (2019) (39), we used data from November to April and averaged over all available years. For Nus et al. (2025) (40), the model data is limited to the period from July to December, and we therefore use the available period for comparison. All computed results are summarized in Table S12.

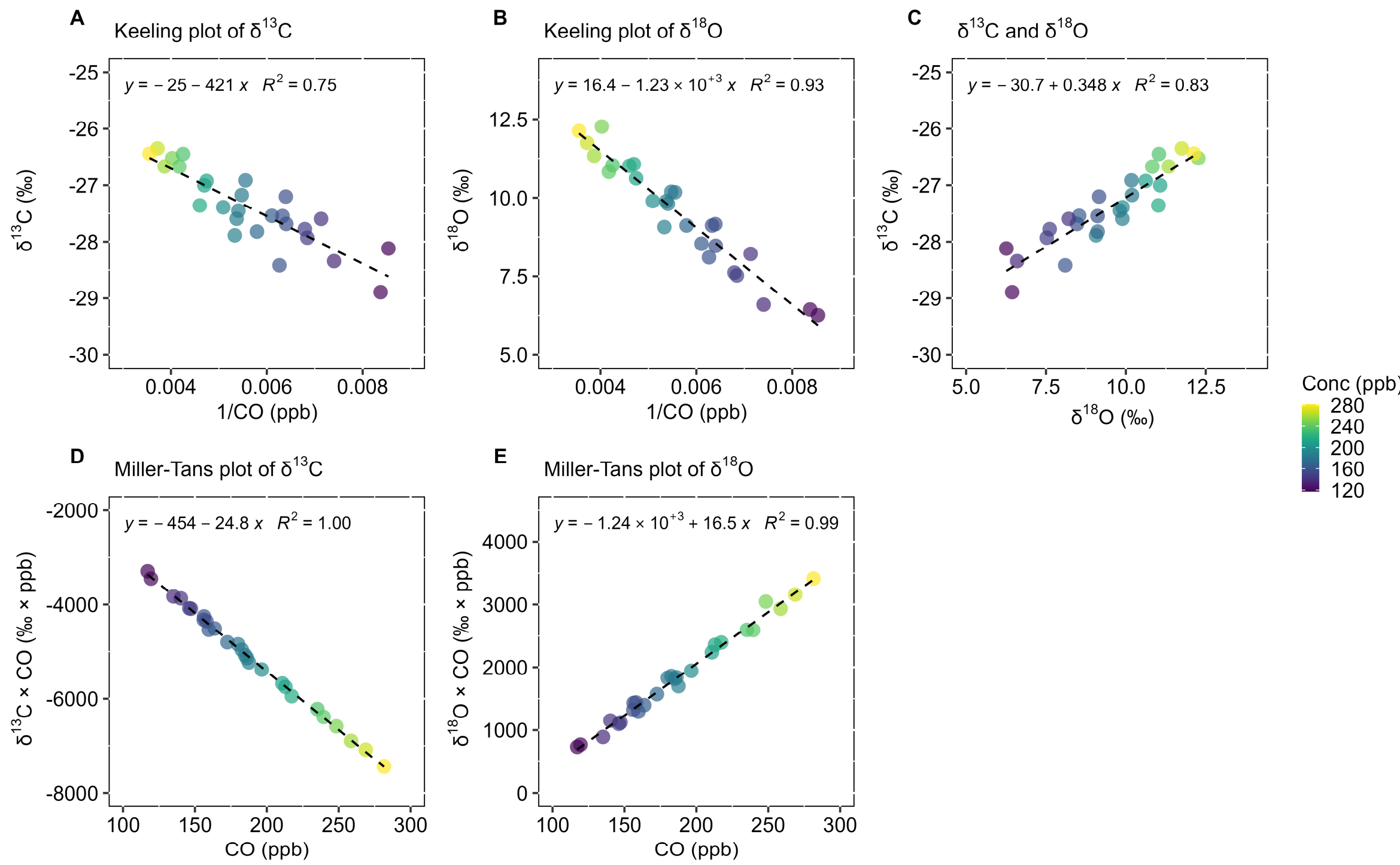

Fig. S1. Mixing ratios and isotopic composition of CO in MCOH during the winter of SAPOEX-2018 (2017–2018). (**A**) Keeling plot for $\delta^{13}C$. (**B**) Keeling plot for $\delta^{18}O$. (**C**) Synchronous variations in $\delta^{13}C$ and $\delta^{18}O$ of CO. (**D**) Miller-Tans plot for $\delta^{13}C$. (**E**) Miller-Tans plot for $\delta^{18}O$.

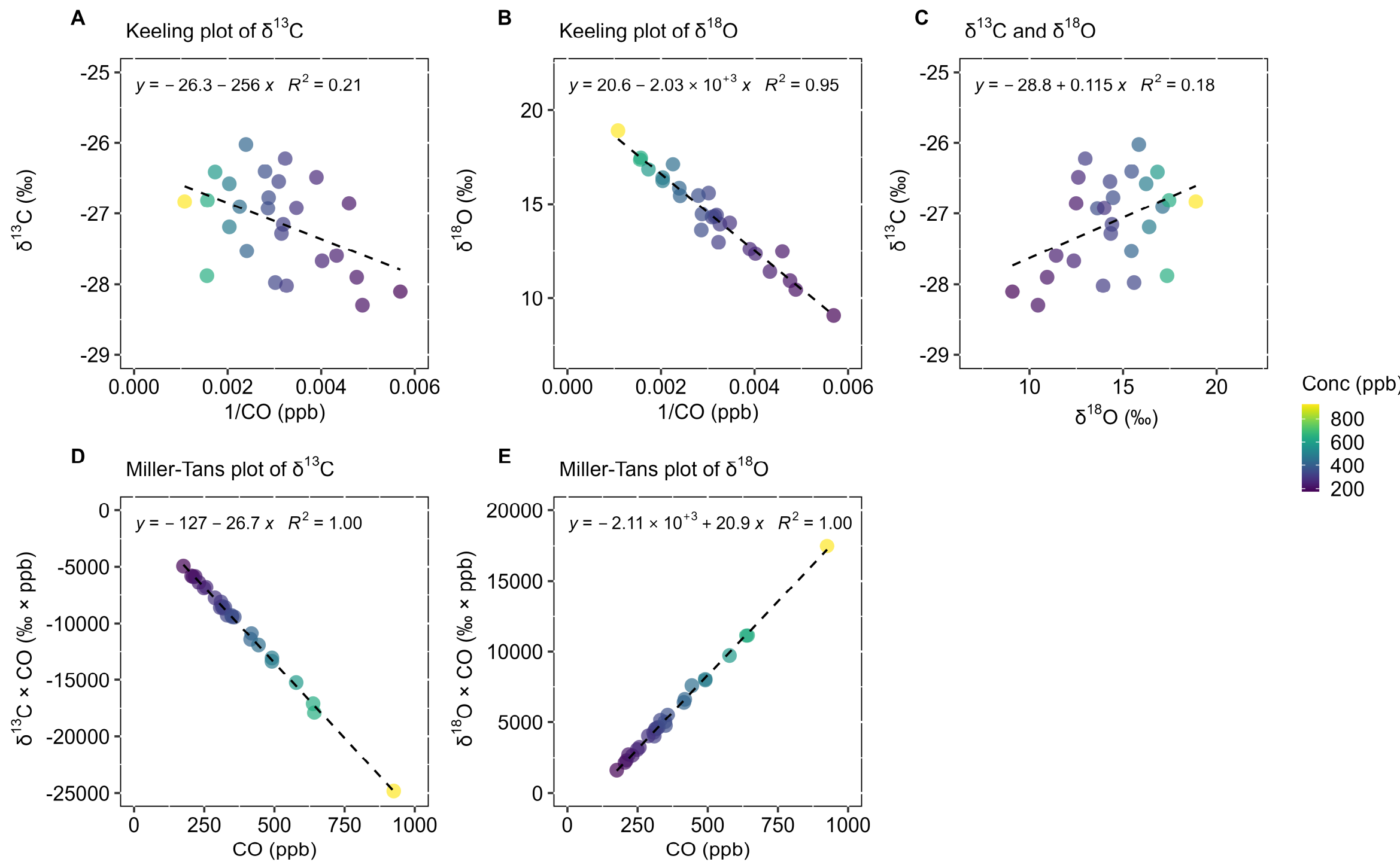

Fig. S2. Mixing ratios and isotopic composition of CO in BCOB during the winter of SAPOEX-2019 (2018–2019). (**A**) Keeling plot for $\delta^{13}C$. (**B**) Keeling plot for $\delta^{18}O$. (**C**) Synchronous variations in $\delta^{13}C$ and $\delta^{18}O$ of CO. (**D**) Miller-Tans plot for $\delta^{13}C$. (**E**) Miller-Tans plot for $\delta^{18}O$.

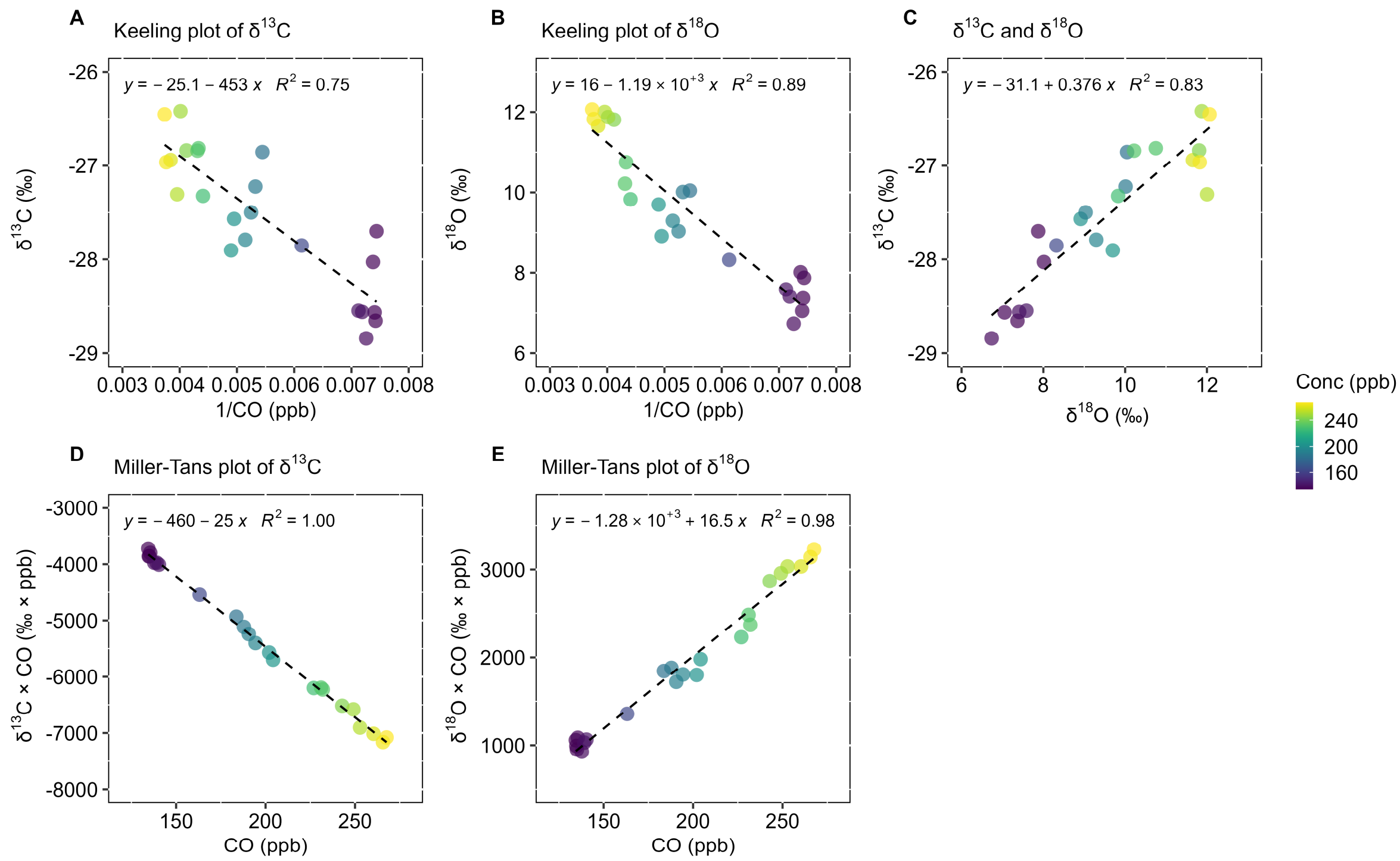

Fig. S3. Mixing ratios and isotopic composition of CO in MCOH during the winter of SAPOEX-2019 (2018–2019). (**A**) Keeling plot for δ^{13}C. (**B**) Keeling plot for δ^{18}O. (**C**) Synchronous variations in δ^{13}C and δ^{18}O of CO. (**D**) Miller-Tans plot for δ^{13}C. (**E**) Miller-Tans plot for δ^{18}O.

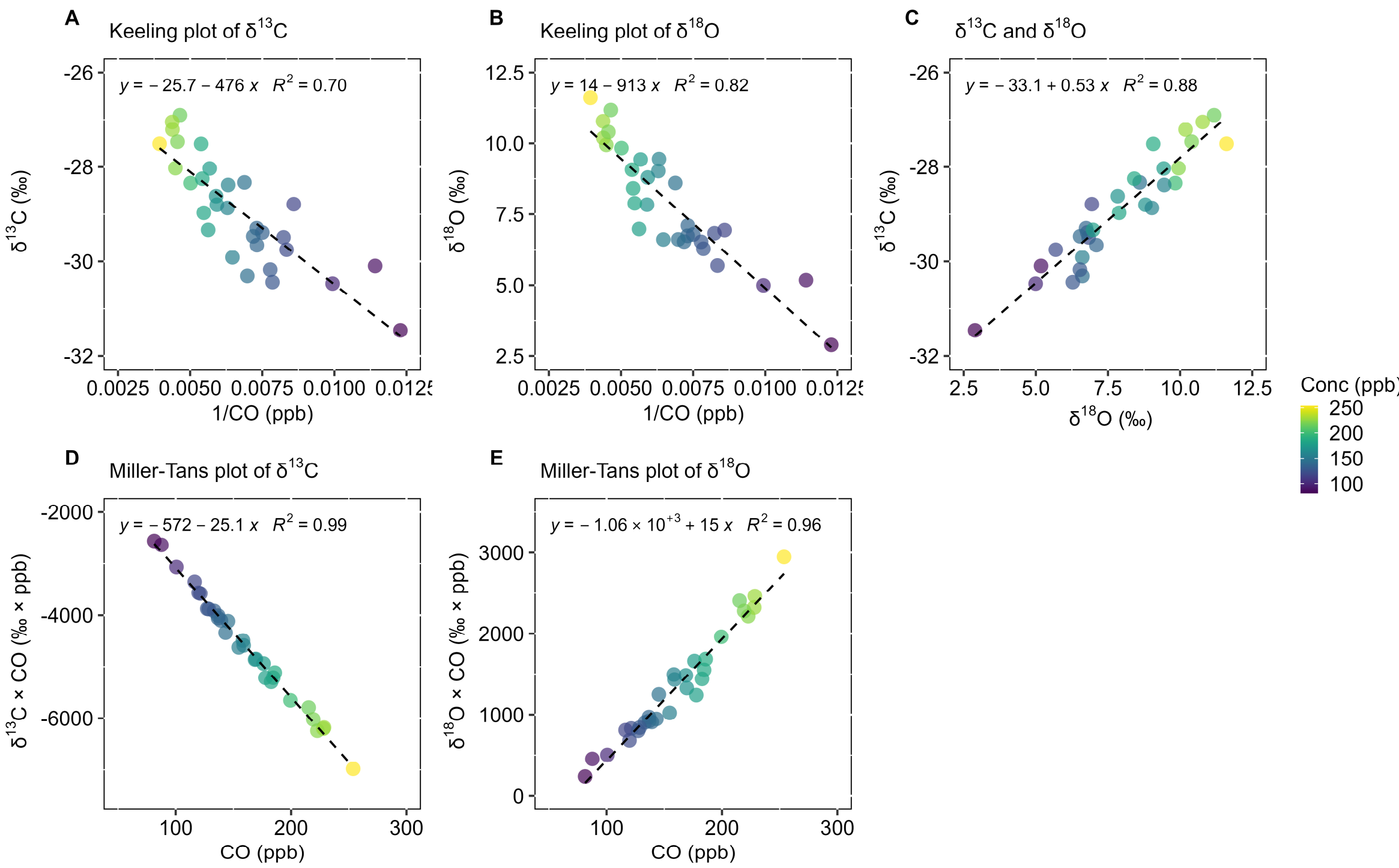

Fig. S4. Mixing ratios and isotopic composition of CO in MCOH during the winter of SAPOEX-2021 (2020–2021). (**A**) Keeling plot for $\delta^{13}C$. (**B**) Keeling plot for $\delta^{18}O$. (**C**) Synchronous variations in $\delta^{13}C$ and $\delta^{18}O$ of CO. (**D**) Miller-Tans plot for $\delta^{13}C$. (**E**) Miller-Tans plot for $\delta^{18}O$.

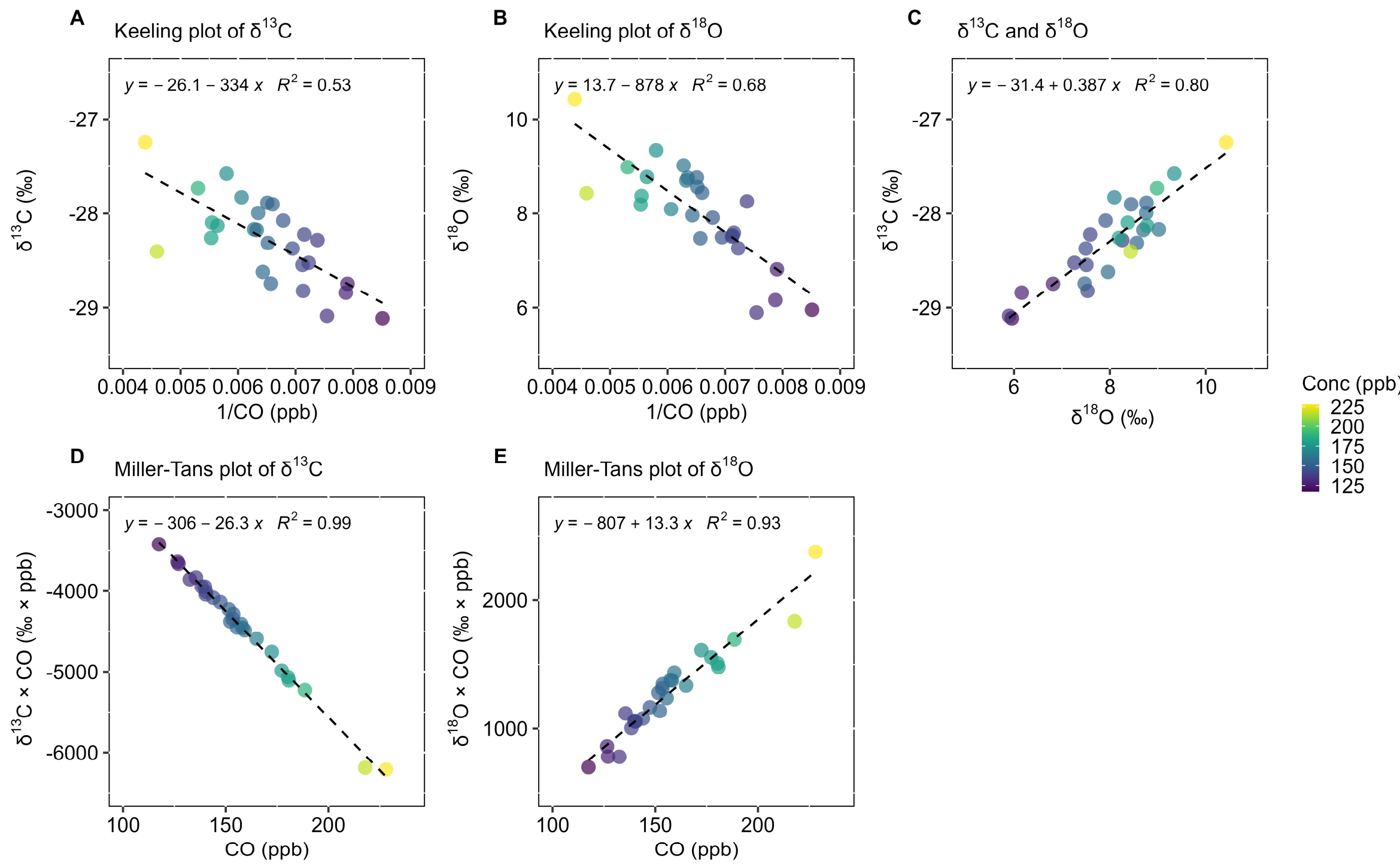

Fig. S5. Mixing ratios and isotopic composition of CO in MCOH during the winter of SAPOEX-2022 (2021–2022). (**A**) Keeling plot for δ^{13}C. (**B**) Keeling plot for δ^{18}O. (**C**) Synchronous variations in δ^{13}C and δ^{18}O of CO. (**D**) Miller-Tans plot for δ^{13}C. (**E**) Miller-Tans plot for δ^{18}O.

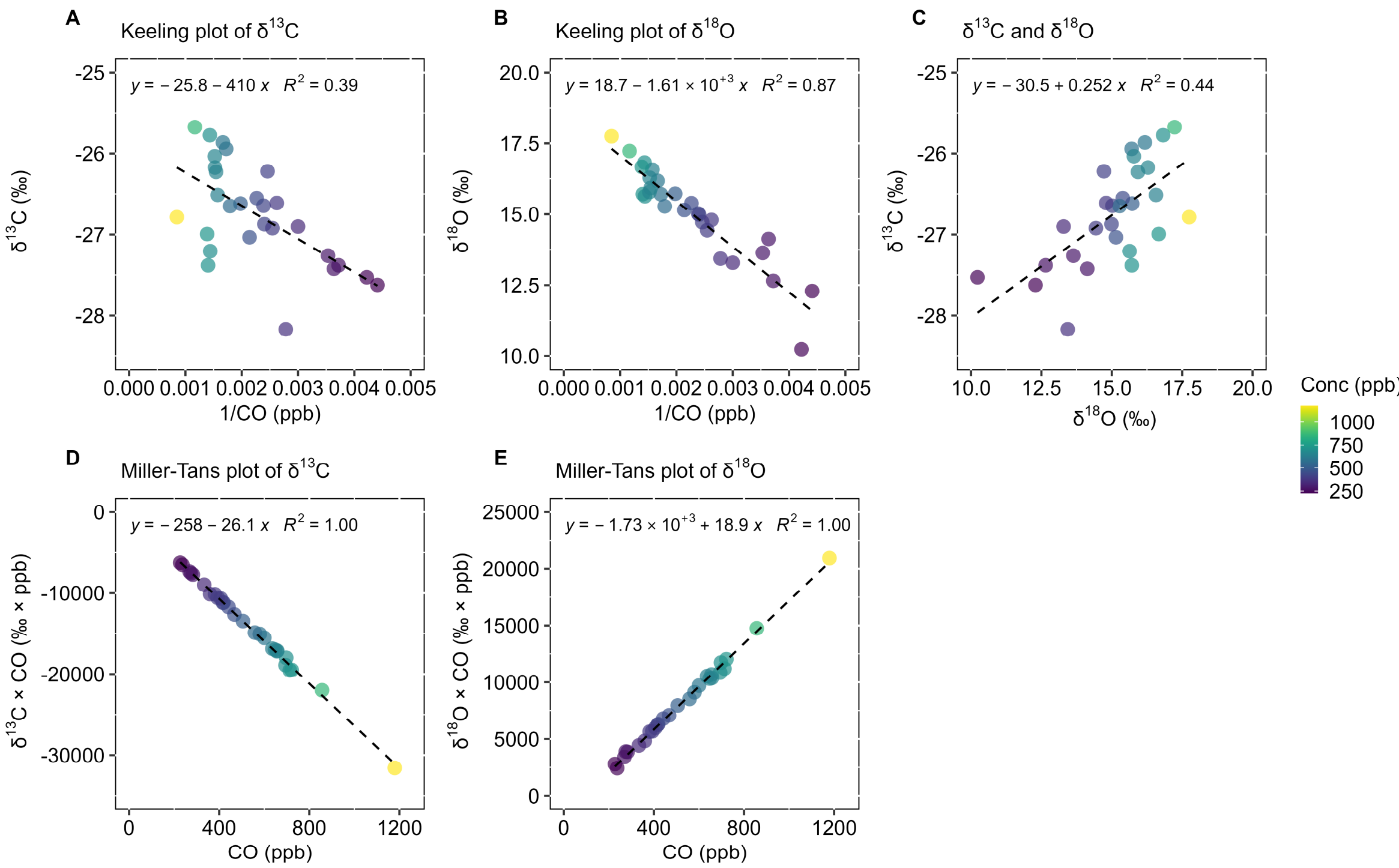

Fig. S6. Mixing ratios and isotopic composition of CO in BCOB during the winter of SAPOEX-2023 (2022–2023). (**A**) Keeling plot for δ^{13}C. (**B**) Keeling plot for δ^{18}O. (**C**) Synchronous variations in δ^{13}C and δ^{18}O of CO. (**D**) Miller-Tans plot for δ^{13}C. (**E**) Miller-Tans plot for δ^{18}O.

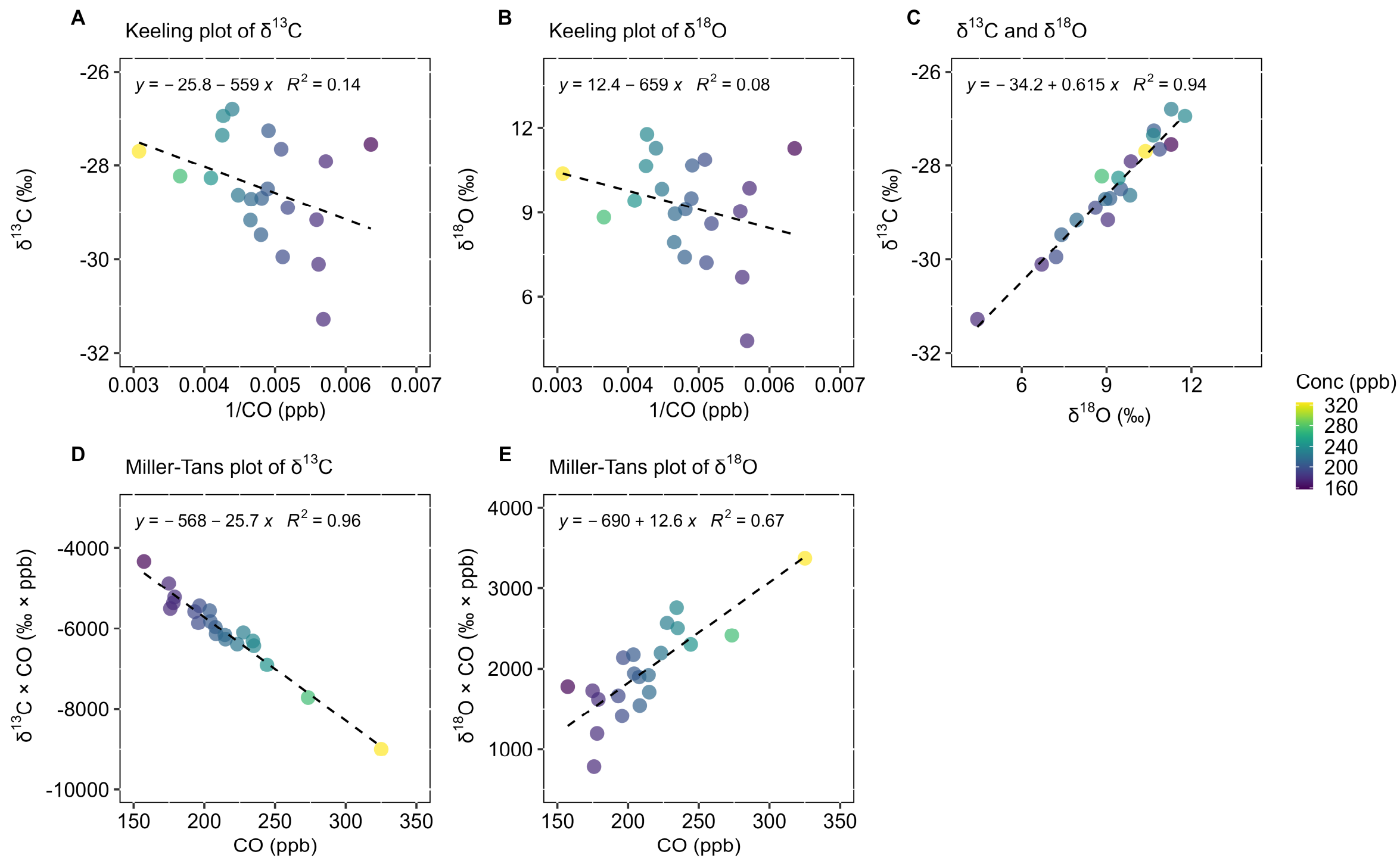

Fig. S7. Mixing ratios and isotopic composition of CO in MCOH during the winter of SAPOEX-2023 (2022–2023). (**A**) Keeling plot for $\delta^{13}C$. (**B**) Keeling plot for $\delta^{18}O$. (**C**) Synchronous variations in $\delta^{13}C$ and $\delta^{18}O$ of CO. (**D**) Miller-Tans plot for $\delta^{13}C$. (**E**) Miller-Tans plot for $\delta^{18}O$.

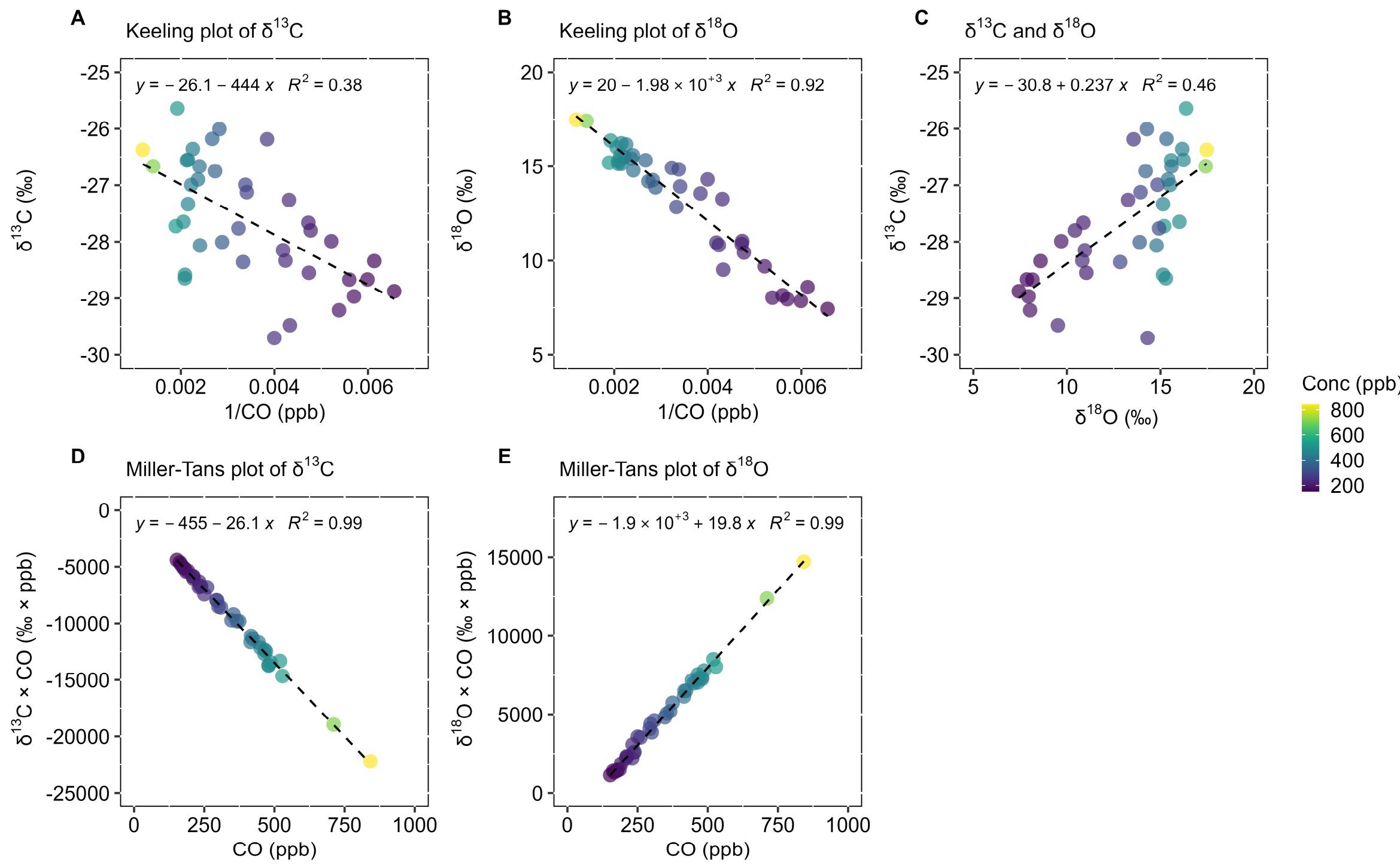

Fig. S8. Mixing ratios and isotopic composition of CO in BCOB during the winter of SAPOEX-2024 (2023–2024). (**A**) Keeling plot for δ^{13}C. (**B**) Keeling plot for δ^{18}O. (**C**) Synchronous variations in δ^{13}C and δ^{18}O of CO. (**D**) Miller-Tans plot for δ^{13}C. (**E**) Miller-Tans plot for δ^{18}O.

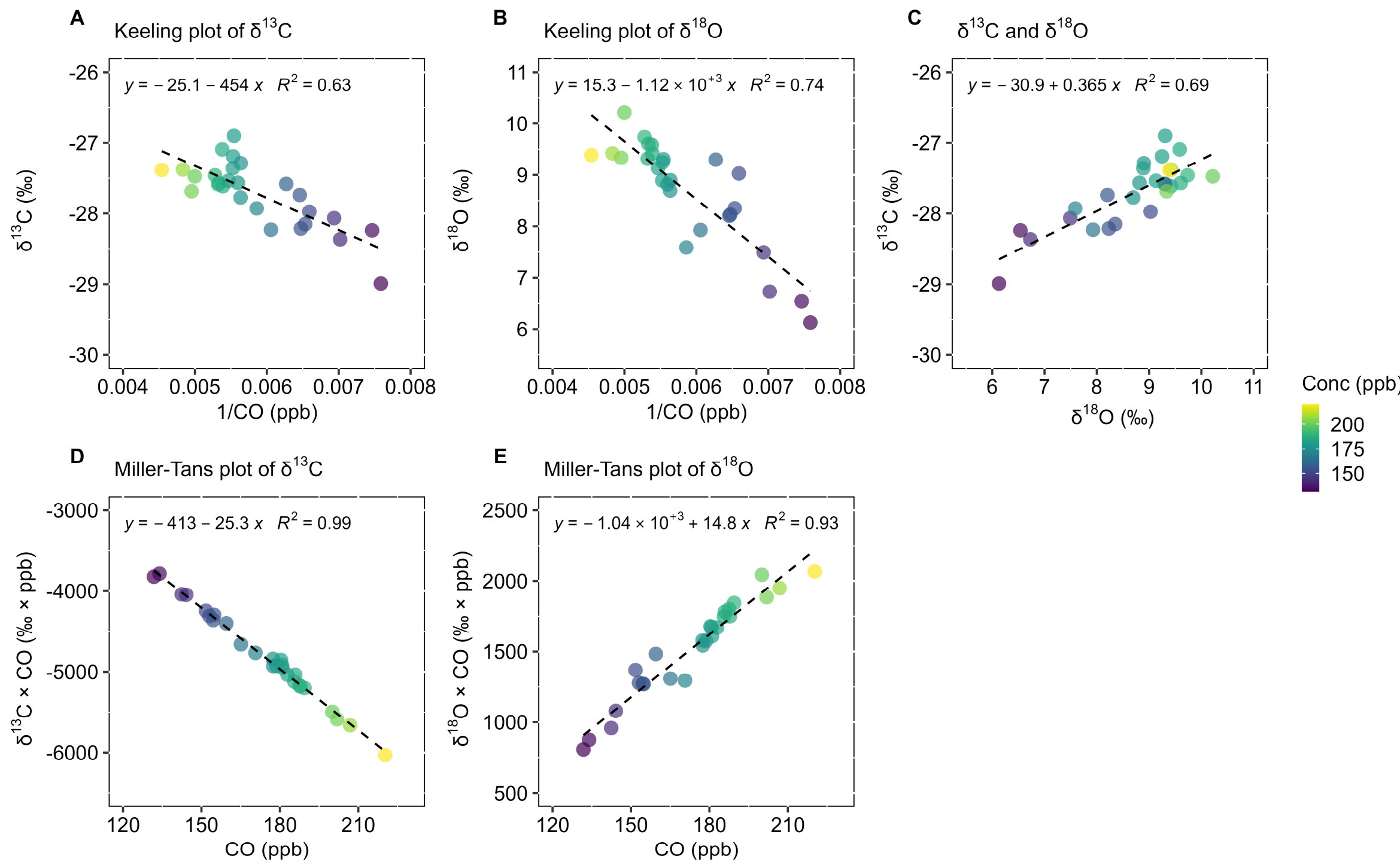

Fig. S9. Mixing ratios and isotopic composition of CO in MCOH during the winter of SAPOEX-2024 (2023–2024). (**A**) Keeling plot for δ^{13}C. (**B**) Keeling plot for δ^{18}O. (**C**) Synchronous variations in δ^{13}C and δ^{18}O of CO. (**D**) Miller-Tans plot for δ^{13}C. (**E**) Miller-Tans plot for δ^{18}O.

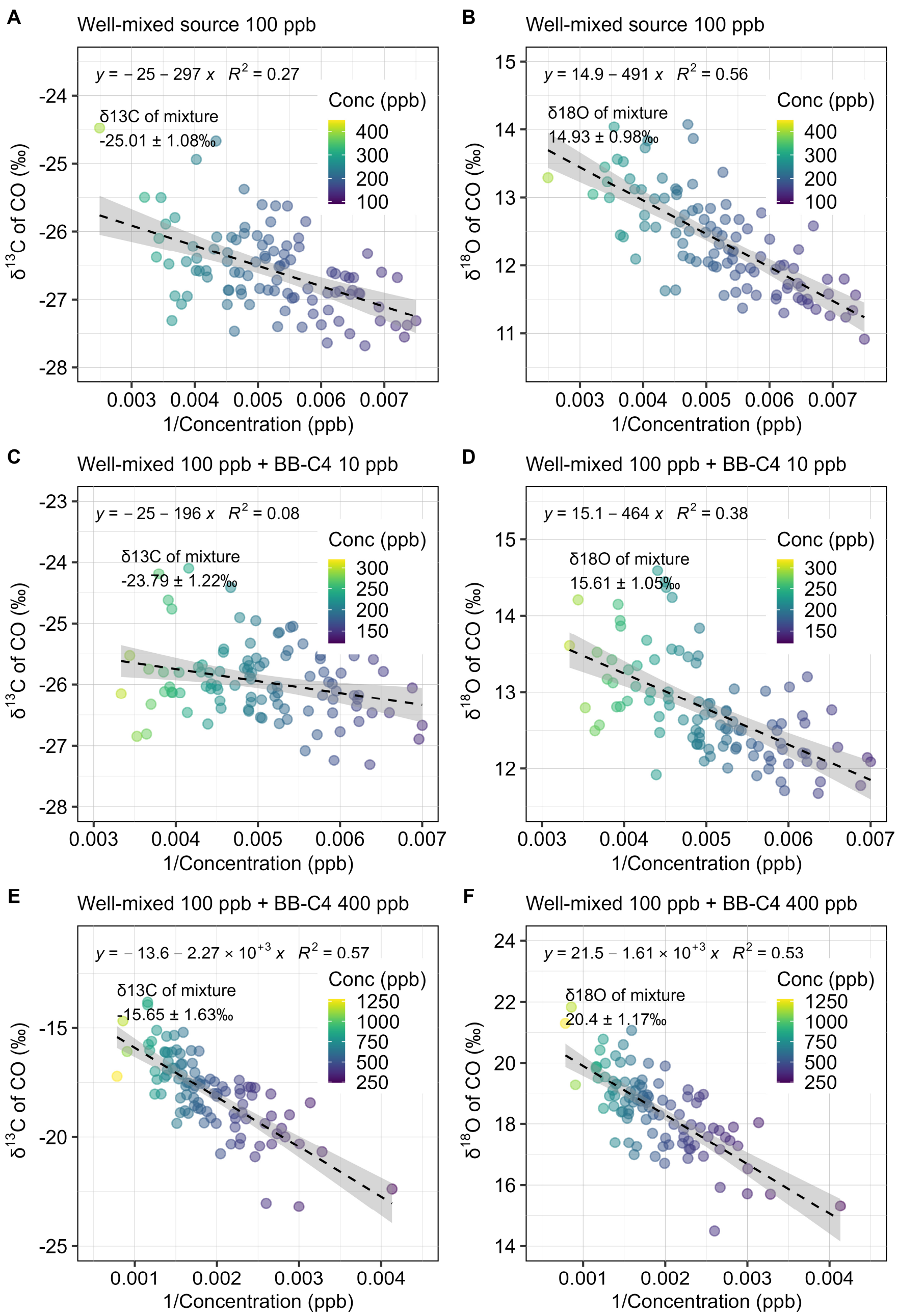

Fig. S10. Interpretation of Keeling plots with contributions from a regional well-mixed source and a local biomass burning source at a background site. All scenarios assume a 100 ppb CO background and synchronous mixing. (**A–B**) Only the regional well-mixed source (100 ppb) is present. (**C–D**) Regional well-mixed source (100 ppb) plus a weak local biomass burning

source (10 ppb, C4 vegetation). (**E–F**) Regional well-mixed source (100 ppb) plus a strong local biomass burning source (400 ppb, C4 vegetation).

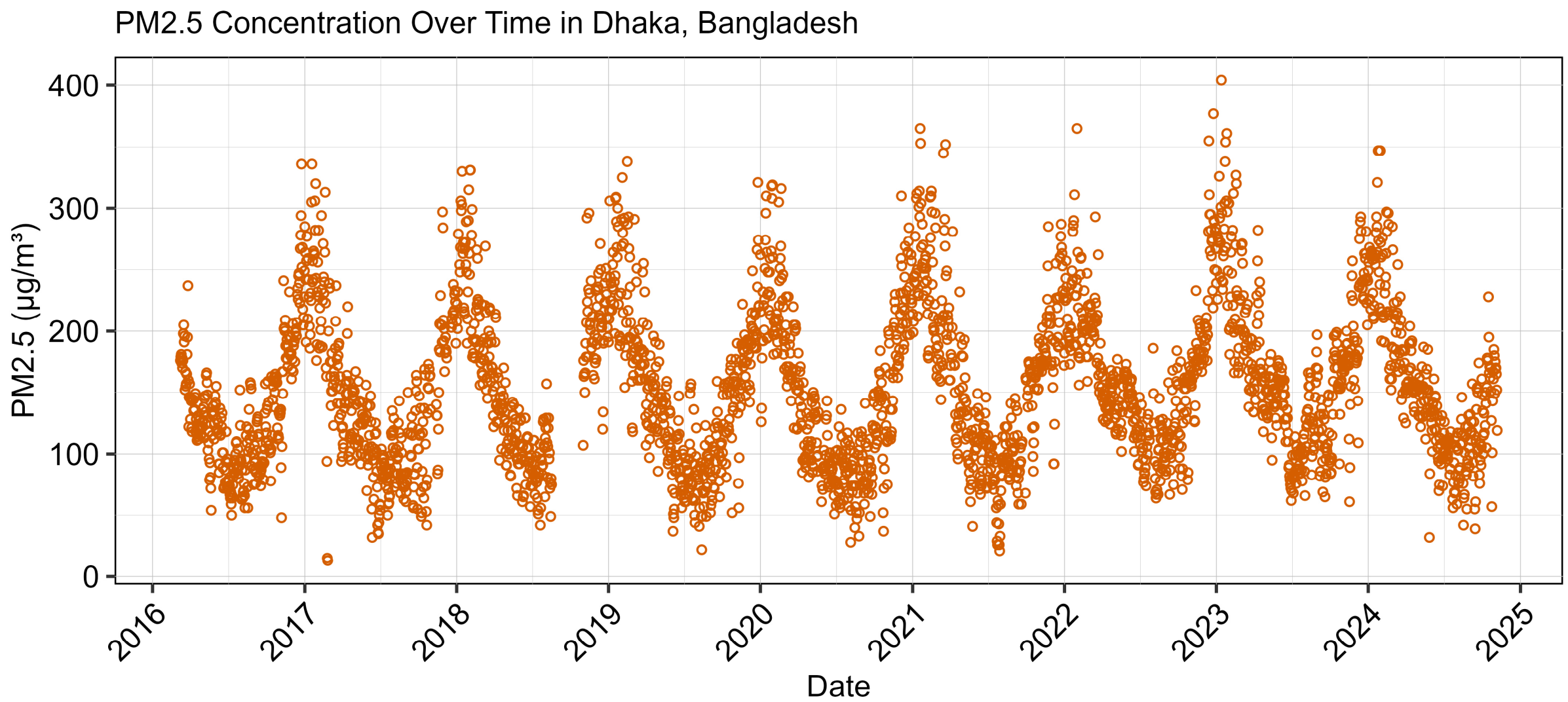

Fig. S11. Observed atmospheric $PM_{2.5}$ concentrations in Dhaka, Bangladesh, from 2016 to 2024. Data sourced from http://aqicn.org.

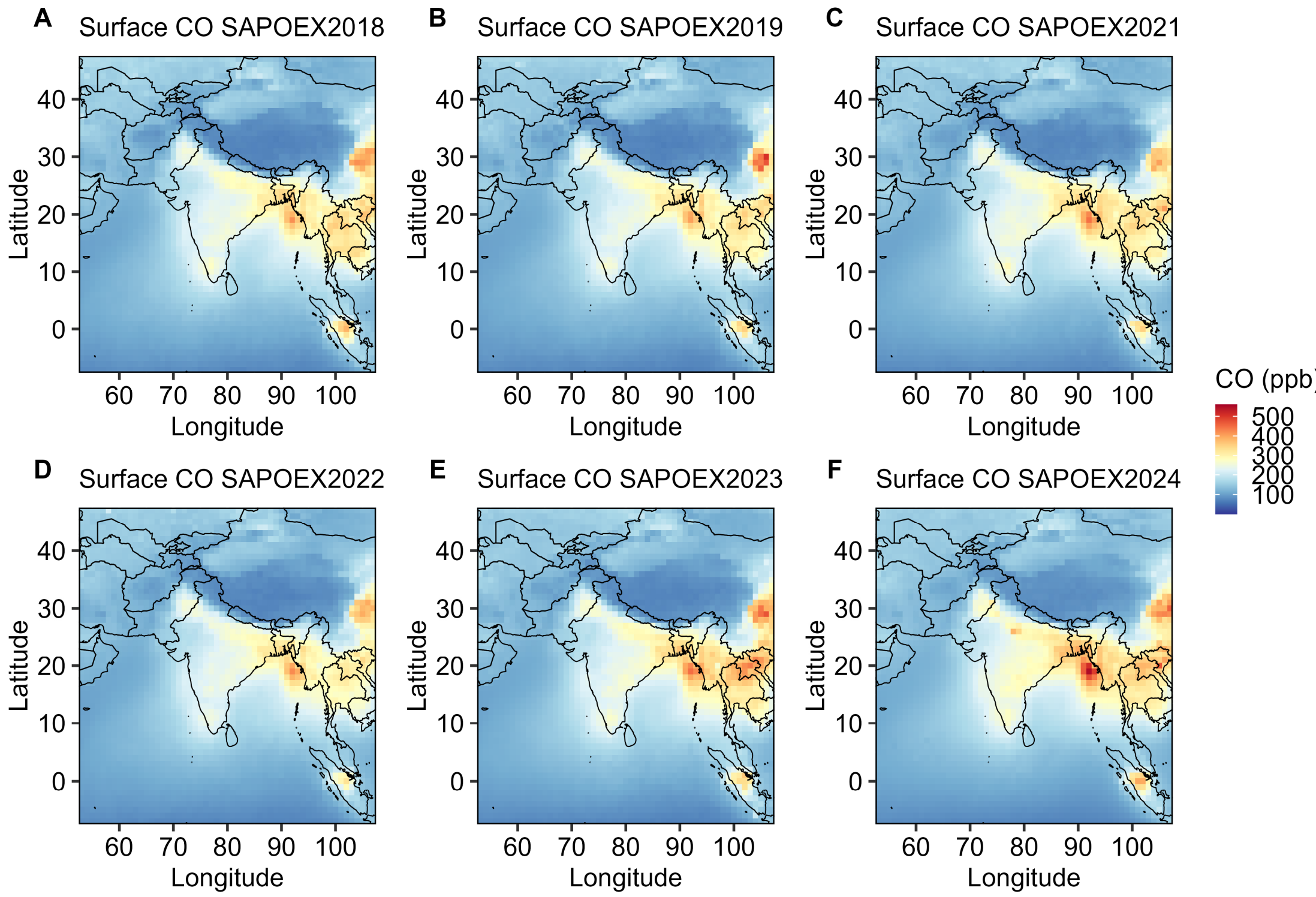

Fig. S12. Monthly mean maps of retrieved surface CO mixing ratio (daytime, 1.0°) over South Asia during winter 2018–2024. Data are from MOPITT MOP03JM v009 (ppbv). (**A**) SAPOEX-2018. (**B**) SAPOEX-2019. (**C**) SAPOEX-2021. (**D**) SAPOEX-2022. (**E**) SAPOEX-2023. (**F**) SAPOEX-2024.

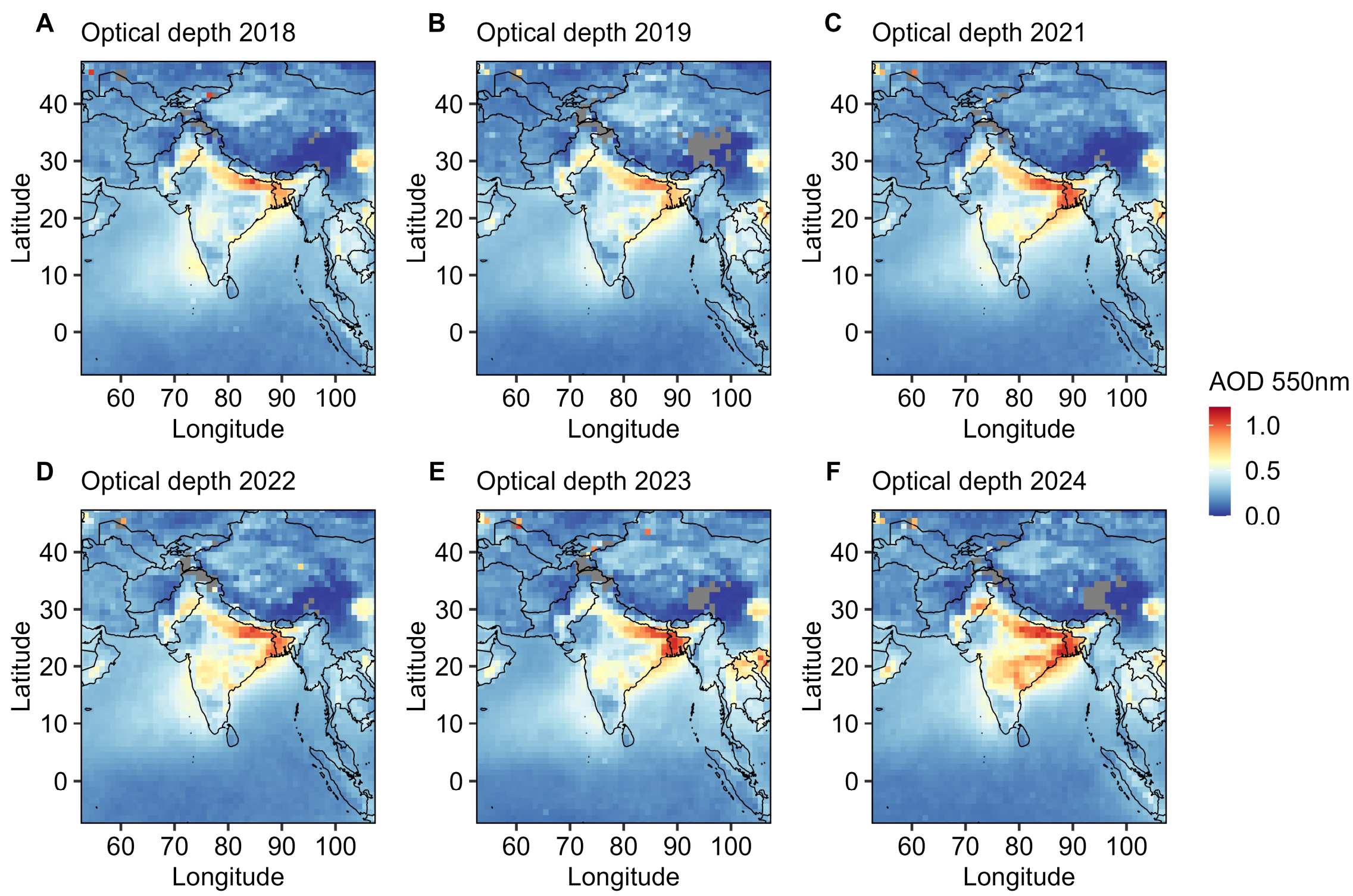

Fig. S13. Wintertime mean AOD (550 nm) over land and ocean in South Asia (2018–2024), based on MODIS-Terra MOD08_M3 v6.1 Dark Target and Deep Blue combined product (1° monthly resolution). (**A**) SAPOEX-2018. (**B**) SAPOEX-2019. (**C**) SAPOEX-2021. (**D**) SAPOEX-2022. (**E**) SAPOEX-2023. (**F**) SAPOEX-2024.

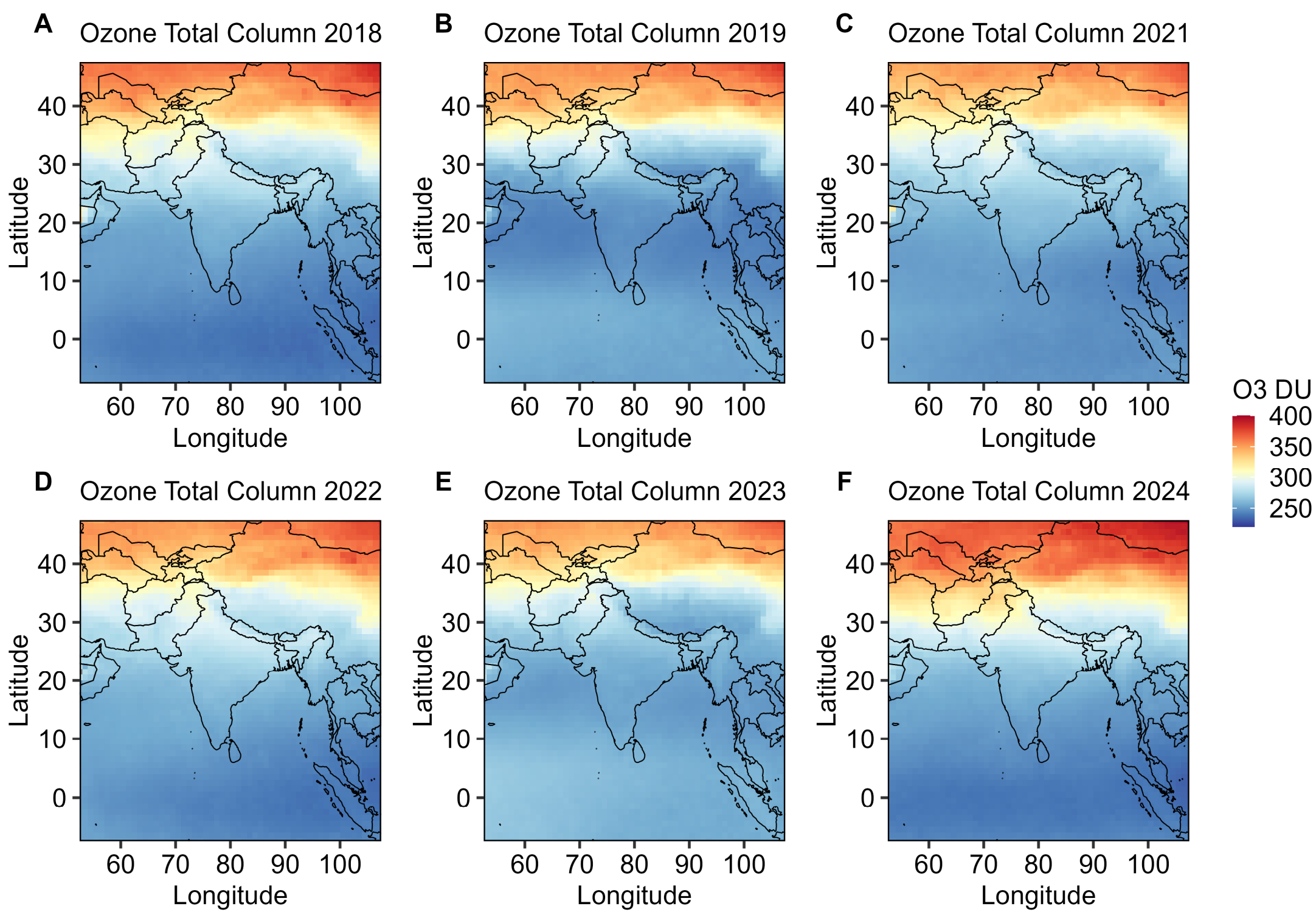

Fig. S14. Wintertime mean total column ozone (daytime/ascending) over South Asia (2018–2024), derived from AIRS AIRS3STM v7.0 (1° monthly resolution). (**A**) SAPOEX-2018. (**B**) SAPOEX-2019. (**C**) SAPOEX-2021. (**D**) SAPOEX-2022. (**E**) SAPOEX-2023. (**F**) SAPOEX-2024.

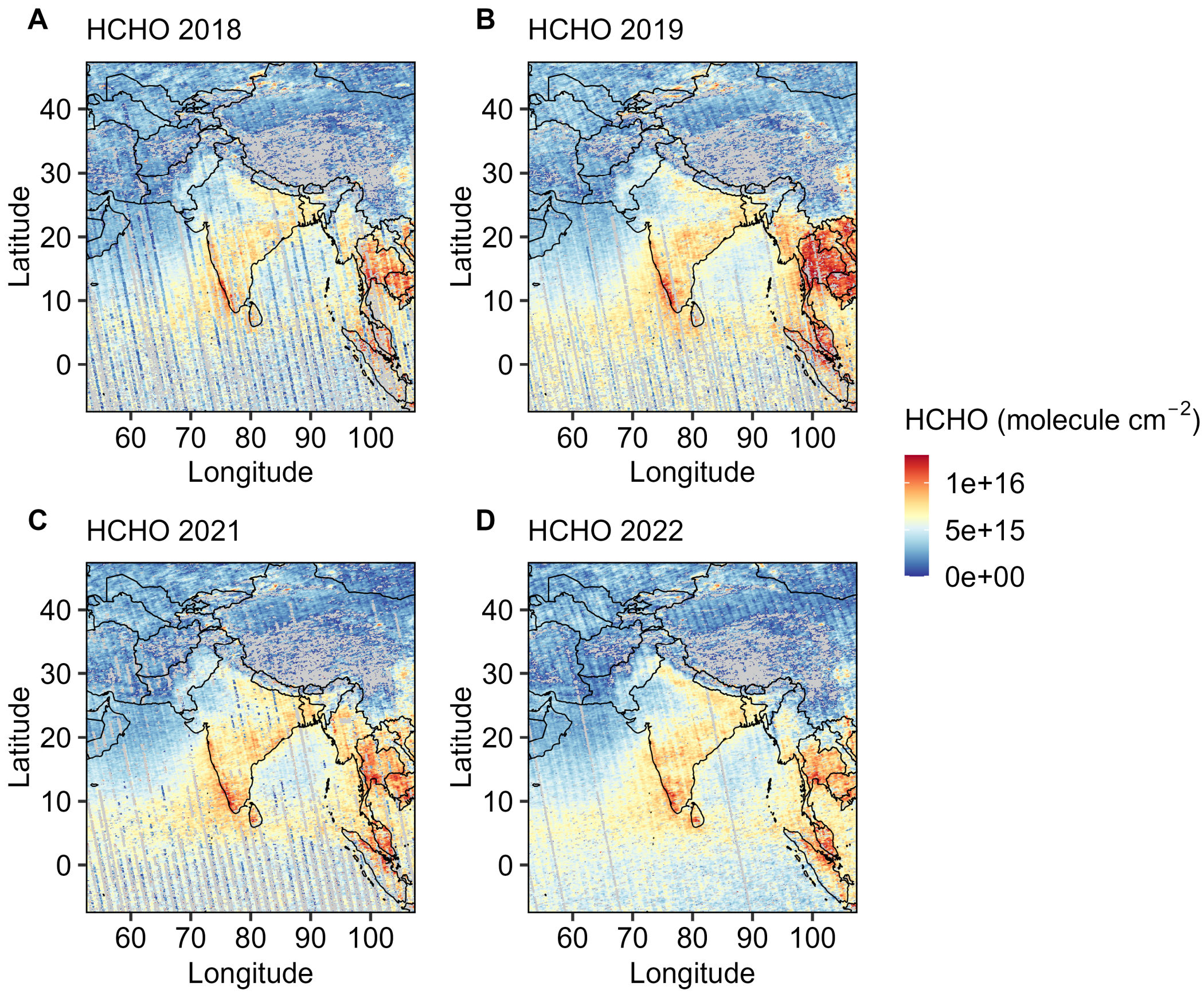

Fig. S15. Wintertime mean formaldehyde (HCHO) column density over South Asia (2018–2022), derived from OMI OMHCHOd v003 (daily, 0.1° resolution). (**A**) SAPOEX-2018. (**B**) SAPOEX-2019. (**C**) SAPOEX-2021. (**D**) SAPOEX-2022.

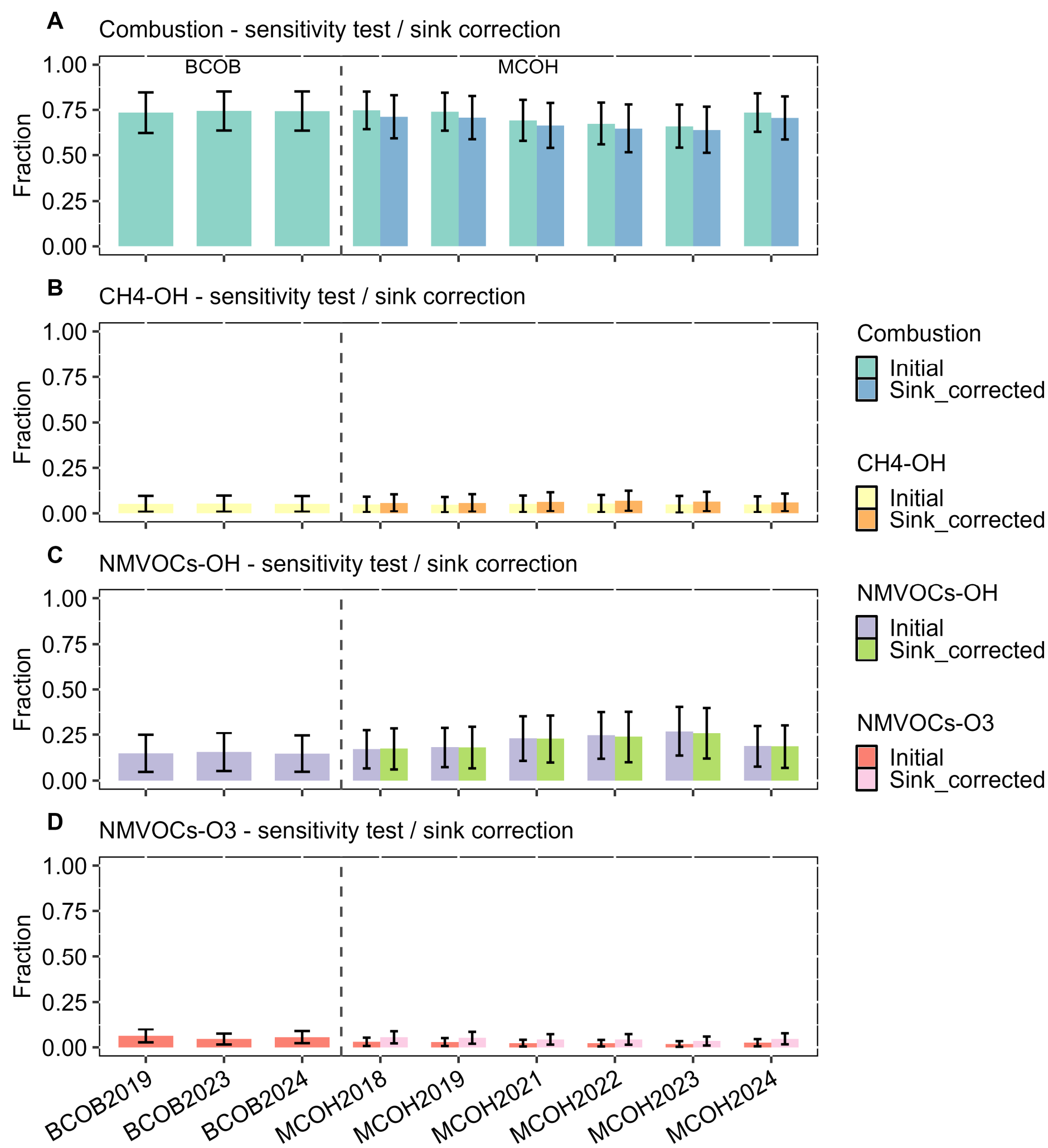

Fig. S16. Sensitivity analysis of dual-isotope CO source apportionment at BCOB and MCOH across multiple years, with and without sink correction. (**A**) Combustion; (**B**) CH_4–OH; (**C**) NMVOCs–OH; (**D**) NMVOCs–O_3. BCOB, located in the source region, does not require sink correction, so the Initial apportionment represents the accurate result. In contrast, MCOH, located over the Indian Ocean and subject to atmospheric oxidation, requires sink correction. For MCOH, Sink_corrected represents the accurate source apportionment, and Initial is the sensitivity test showing the potential bias if the sink effect were not corrected.

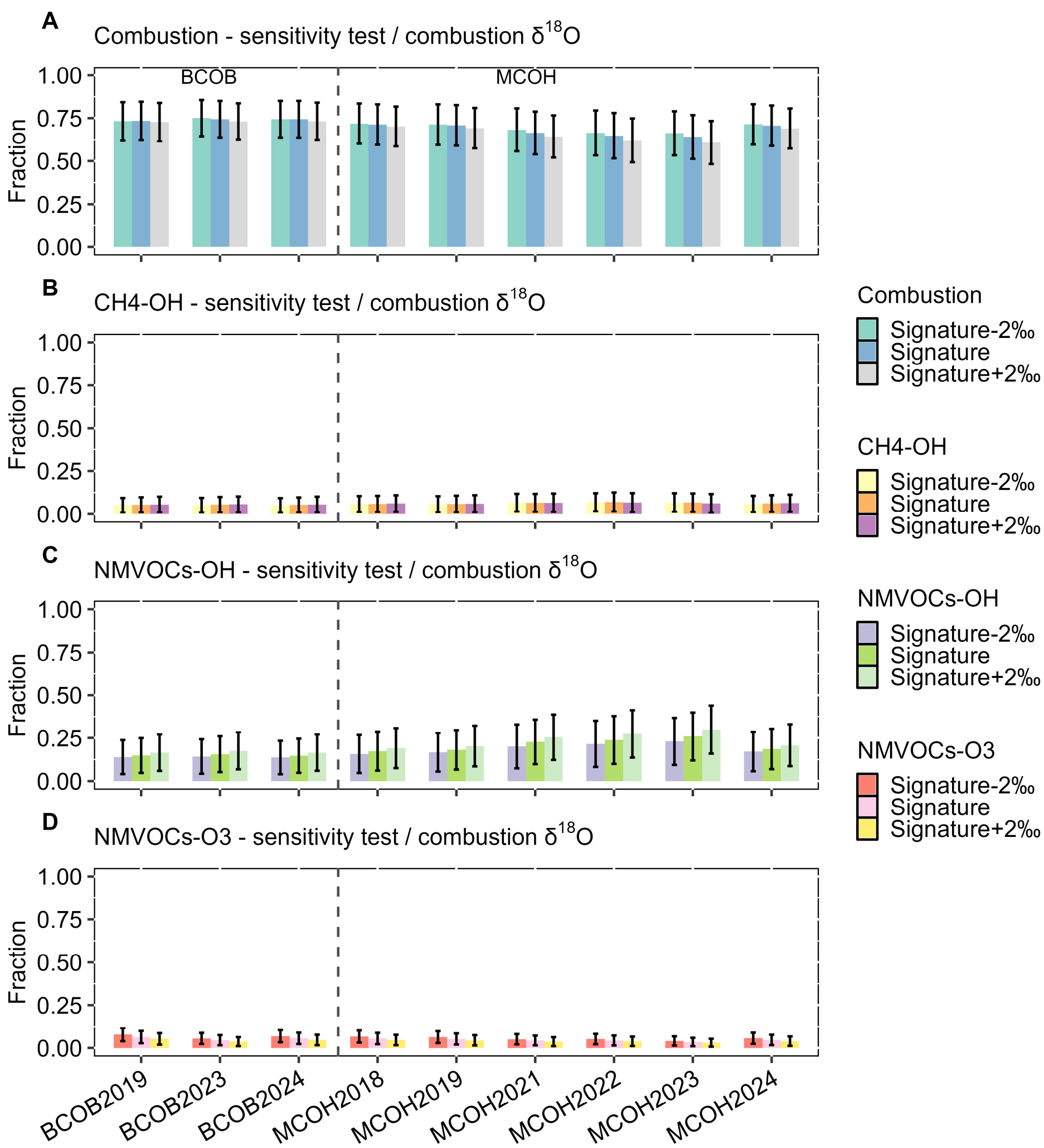

Fig. S17. Sensitivity of dual-isotope CO source apportionment at BCOB and MCOH across multiple years to variations in the combustion $\delta^{18}O$ signature. (**A**) Combustion; (**B**) CH_4–OH; (**C**) NMVOCs–OH; (**D**) NMVOCs–O_3. The mean $\delta^{18}O$ value of the combustion source is perturbed by ±2‰ (with uncertainty unchanged) to assess its influence on source contributions and evaluate the robustness of the apportionment.

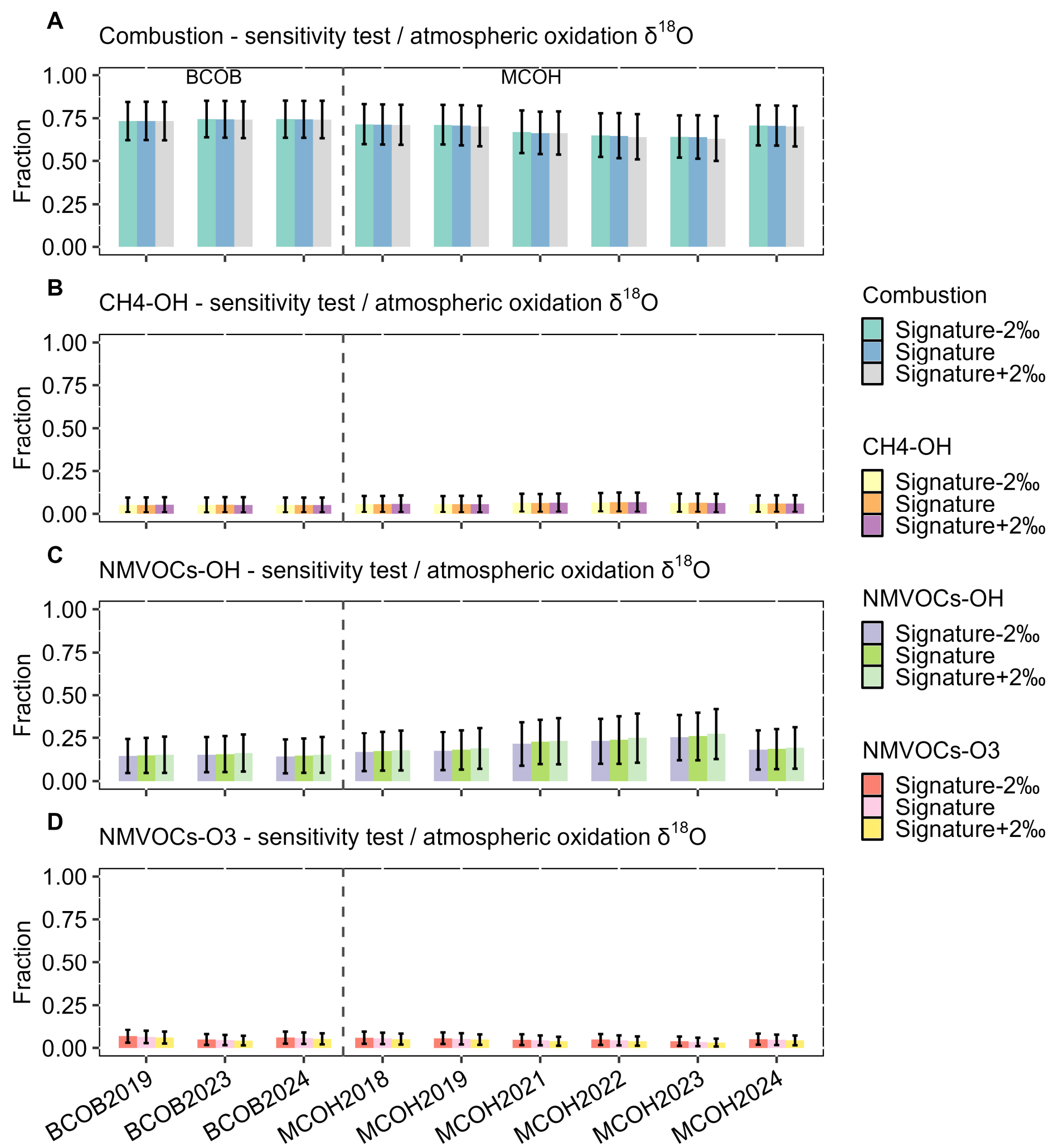

Fig. S18. Sensitivity of dual-isotope CO source apportionment at BCOB and MCOH across multiple years to variations in the atmospheric oxidation δ^{18}O signature. (**A**) Combustion; (**B**) CH_4–OH; (**C**) NMVOCs–OH; (**D**) NMVOCs–O_3. The mean δ^{18}O values of the atmospheric oxidation sources by OH are perturbed by ±2‰ (with uncertainty unchanged) to assess its influence on source contributions and evaluate the robustness of the apportionment.

References

1. P. Yao, *et al.*, ^{13}C signatures of aerosol organic and elemental carbon from major combustion sources in China compared to worldwide estimates. *Sci. Total Environ.* **810**, 151284 (2022).

2. R. Vernooij, *et al.*, Stable carbon isotopic composition of biomass burning emissions – implications for estimating the contribution of C_3 and C_4 plants. *Atmos. Chem. Phys.* **22**, 2871–2890 (2022).

3. B. Gaveau, R. Letolle, M. Monthioux, Evaluation of kinetic parameters from ^{13}C isotopic effect during coal pyrolysis. *Fuel* **66**, 228–231 (1987).

4. S. Dasari, *et al.*, Source Quantification of South Asian Black Carbon Aerosols with Isotopes and Modeling. *Environ. Sci. Technol.* **54**, 11771–11779 (2020).

5. C. M. Stevens, A. F. Wagner, The Role of Isotope Fractionation Effects in Atmospheric Chemistry. *Zeitschrift für Naturforsch. A* **44**, 376–384 (1989).

6. C. A. M. Brenninkmeijer, Measurement of the abundance of ^{14}CO in the atmosphere and the $^{13}C/^{12}C$ and $^{18}O/^{16}O$ ratio of atmospheric CO with applications in New Zealand and Antarctica. *J. Geophys. Res. Atmos.* **98**, 10595–10614 (1993).

7. C. A. M. Brenninkmeijer, T. Röckmann, Principal factors determining the $^{18}O/^{16}O$ ratio of atmospheric CO as derived from observations in the southern hemispheric troposphere and lowermost stratosphere. *J. Geophys. Res. Atmos.* **102**, 25477–25485 (1997).

8. I. J. Vimont, *et al.*, An improved estimate for the $\delta^{13}C$ and $\delta^{18}O$ signatures of carbon monoxide produced from atmospheric oxidation of volatile organic compounds. *Atmos.*

Chem. Phys. **19**, 8547–8562 (2019).

9. P. D. Quay, *et al.*, Carbon isotopic composition of atmospheric CH_4: Fossil and biomass burning source strengths. *Global Biogeochem. Cycles* **5**, 25–47 (1991).

10. C. A. M. Brenninkmeijer, T. Röckmann, M. Bräunlich, P. Jöckel, P. Bergamaschi, Review of progress in isotope studies of atmospheric carbon monoxide. *Chemosph. - Glob. Chang. Sci.* **1**, 33–52 (1999).

11. G. Saueressig, *et al.*, Carbon 13 and D kinetic isotope effects in the reactions of CH_4 with $O(^1D)$ and OH: New laboratory measurements and their implications for the isotopic composition of stratospheric methane. *J. Geophys. Res. Atmos.* **106**, 23127–23138 (2001).

12. T. Röckmann, *et al.*, Using ^{14}C, ^{13}C, ^{18}O and ^{17}O isotopic variations to provide insights into the high northern latitude surface CO inventory. *Atmos. Chem. Phys.* **2**, 147–159 (2002).

13. R. E. Weston, Oxygen Isotope Effects in the Oxidation of Methane to Carbon Monoxide. *J. Phys. Chem. A* **105**, 1656–1661 (2001).

14. T. Röckmann, C. A. M. Brenninkmeijer, P. Neeb, P. J. Crutzen, Ozonolysis of nonmethane hydrocarbons as a source of the observed mass independent oxygen isotope enrichment in tropospheric CO. *J. Geophys. Res. Atmos.* **103**, 1463–1470 (1998).

15. E. Barkan, B. Luz, High precision measurements of $^{17}O/^{16}O$ and $^{18}O/^{16}O$ ratios in H_2O. *Rapid Commun. Mass Spectrom.* **19**, 3737–3742 (2005).

16. V. Masson-Delmotte, *et al.*, Climate change 2021: the physical science basis. *Contrib. Work. Gr. I to sixth Assess. Rep. Intergov. panel Clim. Chang.* **2** (2021).

17. C. A. M. Brenninkmeijer, D. C. Lowe, M. R. Manning, R. J. Sparks, P. F. J. van Velthoven, The ^{13}C, ^{14}C, and ^{18}O isotopic composition of CO, CH_4, and CO_2 in the higher southern latitudes lower stratosphere. *J. Geophys. Res. Atmos.* **100**, 26163–26172 (1995).

18. C. Arsene, A. Bougiatioti, M. Kanakidou, B. Bonsang, N. Mihalopoulos, Tropospheric OH and Cl levels deduced from non-methane hydrocarbon measurements in a marine site. *Atmos. Chem. Phys.* **7**, 4661–4673 (2007).

19. X. Wang, *et al.*, The role of chlorine in global tropospheric chemistry. *Atmos. Chem. Phys.* **19**, 3981–4003 (2019).

20. R. Hossaini, *et al.*, A global model of tropospheric chlorine chemistry: Organic versus inorganic sources and impact on methane oxidation. *J. Geophys. Res. Atmos.* **121**, 14,271-14,297 (2016).

21. S. Gromov, C. A. M. Brenninkmeijer, P. Jöckel, A very limited role of tropospheric chlorine as a sink of the greenhouse gas methane. *Atmos. Chem. Phys.* **18**, 9831–9843 (2018).

22. S. Gromov, C. A. M. Brenninkmeijer, P. Jöckel, Uncertainties of fluxes and $^{13}C/^{12}C$ ratios of atmospheric reactive-gas emissions. *Atmos. Chem. Phys.* **17**, 8525–8552 (2017).

23. C. A. Cantrell, *et al.*, Carbon kinetic isotope effect in the oxidation of methane by the hydroxyl radical. *J. Geophys. Res.* **95**, 22455 (1990).

24. J. A. Davidson, *et al.*, Carbon kinetic isotope effect in the reaction of CH_4 with HO. *J. Geophys. Res. Atmos.* **92**, 2195–2199 (1987).

25. F. Rust, C. M. Stevens, Carbon kinetic isotope effect in the oxidation of methane by hydroxyl. *Int. J. Chem. Kinet.* **12**, 371–377 (1980).

26. G. Saueressig, P. Bergamaschi, J. N. Crowley, H. Fischer, G. W. Harris, Carbon kinetic isotope effect in the reaction of CH_4 with Cl atoms. *Geophys. Res. Lett.* **22**, 1225–1228 (1995).

27. K. L. Feilberg, D. W. T. Griffith, M. S. Johnson, C. J. Nielsen, The ^{13}C and D kinetic isotope effects in the reaction of CH_4 with Cl. *Int. J. Chem. Kinet.* **37**, 110–118 (2005).

28. S. C. Tyler, H. O. Ajie, A. L. Rice, R. J. Cicerone, E. C. Tuazon, Experimentally determined kinetic isotope effects in the reaction of CH_4 with Cl: Implications for atmospheric CH_4. *Geophys. Res. Lett.* **27**, 1715–1718 (2000).

29. E. G. Nisbet, *et al.*, Atmospheric Methane: Comparison Between Methane's Record in 2006–2022 and During Glacial Terminations. *Global Biogeochem. Cycles* **37** (2023).

30. S. Basu, *et al.*, Estimating emissions of methane consistent with atmospheric measurements of methane and δ^{13}C of methane. *Atmos. Chem. Phys.* **22**, 15351–15377 (2022).

31. L. Li, *et al.*, Determination of the stable carbon isotopic compositions of 2-methyltetrols for four forest areas in Southwest China: The implications for the δ^{13}C values of atmospheric isoprene and C_3/C_4 vegetation distribution. *Sci. Total Environ.* **678**, 780–792 (2019).

32. J. Rudolph, *et al.*, The stable carbon isotope ratio of biogenic emissions of isoprene and the potential use of stable isotope ratio measurements to study photochemical processing of isoprene in the atmosphere. *J. Atmos. Chem.* **44**, 39–55 (2003).

33. C. M. Stevens, *et al.*, The kinetic isotope effect for carbon and oxygen in the reaction CO + OH. *Int. J. Chem. Kinet.* **12**, 935–948 (1980).

34. T. Röckmann, *et al.*, Mass-Independent Oxygen Isotope Fractionation in Atmospheric CO

as a Result of the Reaction CO + OH. *Science (80-.).* **281**, 544–546 (1998).

35. C. A. Beale, E. M. Buzan, C. D. Boone, P. F. Bernath, Near-global distribution of CO isotopic fractionation in the Earth's atmosphere. *J. Mol. Spectrosc.* **323**, 59–66 (2016).

36. K. L. Feilberg, M. S. Johnson, C. J. Nielsen, Relative rates of reaction of $^{13}C^{16}O$, $^{12}C^{18}O$, $^{12}C^{17}O$ and $^{13}C^{18}O$ with OH and OD radicals. *Phys. Chem. Chem. Phys.* **7**, 2318 (2005).

37. T. Röckmann, C. A. M. Brenninkmeijer, CO and CO_2 isotopic composition in Spitsbergen during the 1995 ARCTOC campaign. *Tellus B* **49**, 455–465 (1997).

38. Z. Jiang, *et al.*, A 15-year record of CO emissions constrained by MOPITT CO observations. *Atmos. Chem. Phys.* **17**, 4565–4583 (2017).

39. B. Zheng, *et al.*, Global atmospheric carbon monoxide budget 2000–2017 inferred from multi-species atmospheric inversions. *Earth Syst. Sci. Data* **11**, 1411–1436 (2019).

40. J. R. Nüß, *et al.*, Top-down CO emission estimates using TROPOMI CO data in the TM5-4DVAR (r1258) inverse modeling suit. *Geosci. Model Dev.* **18**, 2861–2890 (2025).
